# Thermoprotection by a cell membrane–localized metacaspase in a green alga

**DOI:** 10.1093/plcell/koad289

**Published:** 2023-11-16

**Authors:** Yong Zou, Igor Sabljić, Natalia Horbach, Adrian N Dauphinee, Anna Åsman, Lucia Sancho Temino, Elena A Minina, Marcin Drag, Simon Stael, Marcin Poreba, Jerry Ståhlberg, Peter V Bozhkov

**Affiliations:** Department of Molecular Sciences, Uppsala BioCenter, Swedish University of Agricultural Sciences and Linnean Center for Plant Biology, SE-756 51 Uppsala, Sweden; Department of Molecular Sciences, Uppsala BioCenter, Swedish University of Agricultural Sciences and Linnean Center for Plant Biology, SE-756 51 Uppsala, Sweden; Department of Chemical Biology and Bioimaging, Wroclaw University of Science and Technology, 50-370 Wroclaw, Poland; Department of Molecular Sciences, Uppsala BioCenter, Swedish University of Agricultural Sciences and Linnean Center for Plant Biology, SE-756 51 Uppsala, Sweden; Department of Molecular Sciences, Uppsala BioCenter, Swedish University of Agricultural Sciences and Linnean Center for Plant Biology, SE-756 51 Uppsala, Sweden; Department of Molecular Sciences, Uppsala BioCenter, Swedish University of Agricultural Sciences and Linnean Center for Plant Biology, SE-756 51 Uppsala, Sweden; Department of Molecular Sciences, Uppsala BioCenter, Swedish University of Agricultural Sciences and Linnean Center for Plant Biology, SE-756 51 Uppsala, Sweden; Department of Chemical Biology and Bioimaging, Wroclaw University of Science and Technology, 50-370 Wroclaw, Poland; Department of Molecular Sciences, Uppsala BioCenter, Swedish University of Agricultural Sciences and Linnean Center for Plant Biology, SE-756 51 Uppsala, Sweden; Department of Chemical Biology and Bioimaging, Wroclaw University of Science and Technology, 50-370 Wroclaw, Poland; Department of Molecular Sciences, Uppsala BioCenter, Swedish University of Agricultural Sciences and Linnean Center for Plant Biology, SE-756 51 Uppsala, Sweden; Department of Molecular Sciences, Uppsala BioCenter, Swedish University of Agricultural Sciences and Linnean Center for Plant Biology, SE-756 51 Uppsala, Sweden

## Abstract

Caspases are restricted to animals, while other organisms, including plants, possess metacaspases (MCAs), a more ancient and broader class of structurally related yet biochemically distinct proteases. Our current understanding of plant MCAs is derived from studies in streptophytes, and mostly in Arabidopsis (*Arabidopsis thaliana*) with 9 MCAs with partially redundant activities. In contrast to streptophytes, most chlorophytes contain only 1 or 2 uncharacterized MCAs, providing an excellent platform for MCA research. Here we investigated CrMCA-II, the single type-II MCA from the model chlorophyte Chlamydomonas (*Chlamydomonas reinhardtii*). Surprisingly, unlike other studied MCAs and similar to caspases, CrMCA-II dimerizes both in vitro and in vivo. Furthermore, activation of CrMCA-II in vivo correlated with its dimerization. Most of CrMCA-II in the cell was present as a proenzyme (zymogen) attached to the plasma membrane (PM). Deletion of *CrMCA-II* by genome editing compromised thermotolerance, leading to increased cell death under heat stress. Adding back either wild-type or catalytically dead CrMCA-II restored thermoprotection, suggesting that its proteolytic activity is dispensable for this effect. Finally, we connected the non-proteolytic role of CrMCA-II in thermotolerance to the ability to modulate PM fluidity. Our study reveals an ancient, MCA-dependent thermotolerance mechanism retained by Chlamydomonas and probably lost during the evolution of multicellularity.

## Teaser

A plasma membrane–localized metacaspase mediates membrane fluidity and confers thermotolerance in *Chlamydomonas reinhardtii* independently of its proteolytic activity.

**IN A NUTSHELL**

**Background:** Metacaspases are a large class of proteases spanning all kingdoms of life including bacteria but absent from animals; they are believed to represent an ancestral group from which animal-specific caspases evolved. In contrast to caspases, a textbook example of well-studied proteases, metacaspases remain poorly understood. Recent research has begun to link metacaspases to the biology of land plants, which possess multiple metacaspase genes (e.g. 9 in *Arabidopsis thaliana*) split into 2 major types (I and II), which substantially complicates their characterization. By contrast, green algae represent an ideal model for metacaspase research, as their genomes carry only 1 or 2 metacaspase genes.

**Question:** We wished to establish a toolbox of chemical probes and mutant strains for metacaspase research in a major unicellular green algal model, Chlamydomonas (*Chlamydomonas reinhardtii*). With such a toolbox in hand, we intended to investigate the fundamental functions of metacaspases in a unicellular plant lineage.

**Findings:** Knockout of the single type-II metacaspase gene of Chlamydomonas (named *CrMCA-II*) decreased algal fitness under heat stress (42 °C), resulting in suppressed growth and increased cell death. Unlike other studied plant metacaspases, CrMCA-II was abundantly associated with the plasma membrane but partially translocated to the cytoplasm during heat treatment. Another peculiar feature of CrMCA-II was its oligomerization to form both catalytically active dimers and larger, megadalton-scale complexes comprising inactive proenzyme (zymogen). We discovered that the thermoprotective function of CrMCA-II is independent of its proteolytic activity but instead might be associated with an unknown mechanism by which CrMCA-II mediates fluidization of the plasma membrane.

**Next steps:** Understanding exactly how CrMCA-II works at the subcellular and molecular levels to confer thermoprotection to algal cells awaits further investigations. In this context, isolation and characterization of the megadalton-size CrMCA-II complex, including its structural analysis, represent a particular challenge.

## Introduction

The first caspase, interleukin-1*β*-converting enzyme (ICE), better known as caspase-1, was discovered in 1992 (Cerretti et al. 1992; Thornberry et al. 1992); shortly thereafter, the cell death gene *ced-*3 from the nematode *Caenorhabditis elegans* (Yuan et al. 1993) was identified, heralding a flurry of research on caspases that continues to this day (Julien and Wells 2017; Van Opdenbosch and Lamkanfi 2019; Kesavardhana et al. 2020; Ross et al. 2022). However, caspases are a relatively small and mostly animal-specific part of a large superfamily of cysteine proteases (C14 within the CD clan), all sharing a caspase-like structural fold and present throughout all kingdoms of life (Uren et al. 2000; Minina et al. 2020). Among them, eukaryotic metacaspases (MCAs) and prokaryotic MCA-like proteases form the phylogenetically broadest group, with members found in all living organisms except animals (Tsiatsiani et al. 2011; McLuskey and Mottram 2015; Minina et al. 2017; Klemenčič and Funk 2019). Despite structural similarity between MCAs and caspases, the 2 groups of proteases fundamentally differ in their substrate specificity, with MCAs, unlike Asp-specific caspases, cleaving exclusively after positively charged (Arg or Lys) residues (Vercammen et al. 2004; Sundström et al. 2009; Minina et al. 2020).

Whereas all known eukaryotic MCAs contain both p20- (catalytic) and p10-like conserved regions, MCAs are classified into 3 major types based on the presence of additional protein modules and the relative position of the p20 and p10 regions (Minina et al. 2020). Thus, the distinguishing feature of type-I MCAs is the N-terminal pro-domain. Type-II MCAs have a long linker separating the p20 and p10 regions, whereas type-III MCAs are defined by swapping the order of the 2 regions, with p10 being located N-terminally to the catalytic p20 region. Notably, there is variation in the amino acid sequence, length of distinct protein regions, and the occurrence of additional motifs among MCAs of the same type, in particular for type-I MCAs that is presumably responsible for the differences in their biochemical properties and physiological functions (Klemenčič and Funk 2019). However, further structure–function categorization of the members within each MCA type is not possible today due to limited success in the structural analysis of these enzymes, with only 3 type-I (*Trypanosoma brucei* TbMCA-Ib, also named MCA2; *Saccharomyces cerevisiae* ScMCA-I, also named MCA1 or YCA1; and *Candida glabrata* CgMCA-I) and 2 type-II (AtMCA-IIa, also named MC4; and AtMCA-IIf, also named MC9, *Arabidopsis thaliana*) MCA crystal structures being available (McLuskey et al. 2012; Wong et al. 2012; Zhu et al. 2020; Conchou et al. 2022; Stael et al. 2023).

Among the 3 types of MCAs, type I is found in all nonanimal organisms, while type II is specific for Chloroplastida (green plants) and type III for Heterokontophyta (phytoplanktonic protists; Choi and Berges 2013; Minina et al. 2017; Klemenčič and Funk 2019). Thus, most annotated green plant genomes encode both type-I and type-II MCAs. Green plants form a monophyletic taxon comprising both land plants and green algae redistributed into 2 evolutionary lineages, the Chlorophyta (chlorophytes) and Streptophyta (streptophytes), that diverged over a billion years ago (Bemer 1985; Morris et al. 2018). While the chlorophytes comprise only green algae, the streptophytes include both land plants and the remaining green algae (called “streptophyte algae”; Becker and Marin 2009).

Research on plant MCAs has so far almost exclusively concerned streptophytes and was dominated by the use of Arabidopsis (*A. thaliana*), due to its advantages as a genetically tractable model system with a small genome and having relatively well-understood biology. The Arabidopsis *MCA* family consists of 9 genes, with 3 type-I (*MC1–MC3* or *MCA-Ia–MCA-Ic*, according to original or updated nomenclature, respectively) and 6 type-II (*MC4–MC9* or *MCA-IIa–MCA-IIf*), the latter including 4 tandem duplicated genes (*MC4–MC7* or *MCA-IIa–MCA-IId*; Vercammen et al. 2004; Minina et al. 2020). Arabidopsis MCAs are involved in the regulation of immune and stress responses, aging, and programmed cell death (Coll et al. 2010, 2014; Watanabe and Lam 2011; Bollhöner et al. 2013; Hander et al. 2019; Shen et al. 2019; Wang et al. 2021; Li et al. 2022). For recent reviews on Arabidopsis MCAs, see Minina et al. (2017), Salguero-Linares and Coll (2019), and Huh (2022). The absence of strong developmental or fitness phenotypes seen in single *MCA* knockouts in Arabidopsis points to some degree of redundancy among different MCAs (Tsiatsiani et al. 2011; Huh 2022). Furthermore, while some MCA-dependent proteolytic functions might be isoform-specific, it is not easy to demonstrate so experimentally. Indeed, MCAs have rather loose substrate cleavage specificity for residues in the P2, P3, and P4 positions (Vercammen et al. 2006; Sundström et al. 2009; Tsiatsiani et al. 2013). This loose substrate cleavage specificity complicates ascribing a given physiological response or developmental event to the proteolytic cleavage of a target protein substrate by a particular MCA, since a mixture of MCAs may exhibit similar substrate specificity within a given cell (or cell lysate).

With these arguments in mind, we turned our attention to the chlorophyte lineage, whose sequenced genomes reveal mostly 1 or 2 *MCA* genes (in contrast to several in streptophytes; Tsiatsiani et al. 2011), providing a powerful paradigm for plant MCA research. Despite this obvious advantage, the involvement of MCAs in chlorophyte biology is currently lacking, thus precluding evolutionary insight into the MCA-dependent functions in green plants.

We set out here to establish chemical and genetic toolboxes for exploring MCAs in a chlorophyte, Chlamydomonas (*Chlamydomonas reinhardtii*). Chlamydomonas is a unicellular green alga used as a model organism by virtue of having very few cell types and simplified cell-to-cell communication, compared to Arabidopsis (Gutman and Niyogi 2004), and combining key animal (e.g. cell motility) and plant (e.g. photosynthesis) characteristics (Merchant et al. 2007). The Chlamydomonas genome encodes 1 type-I and 2 type-II MCAs named CrMCA-I and CrMCA-II, respectively, according to the current nomenclature (Minina et al. 2020). Such genetic simplicity renders Chlamydomonas a favorable model for uncovering primordial roles for these 2 types of MCAs. Here we report biochemical and functional studies of CrMCA-II. Unlike other MCAs, CrMCA-II can oligomerize both in vitro and in vivo. It is predominantly present as a zymogen, associated with the plasma membrane (PM), but translocates to the cytoplasm upon heat stress (HS). Genetic experiments combined with live-cell imaging revealed that CrMCA-II plays a protease-independent cytoprotective role under HS, presumably related to its ability to module PM fluidity.

## Results

### Phylogenetic position of Chlamydomonas MCAs

A BLAST search revealed 1 type-I and 2 type-II MCA proteins encoded by the genomes of species in the order Chlamydomonadales, including Chlamydomonas, *Volvox carteri* and *Gonium pectorale*. Some green algae, e.g. *Raphidocelis subcapitata* and *Auxenochlorella protothecoides*, completely lack a type-II *MCA* and contain only type-I *MCA*s. To gain insight into the evolutionary position of Chlamydomonas MCAs, we performed a phylogenetic analysis of MCA protein sequences from members of the green lineage with well-annotated genomes. This analysis demonstrated that both Chlamydomonas MCAs, CrMCA-I and CrMCA-II, as well as MCAs from other chlorophytes, share common ancestors with and are close relatives of MCAs from land plants (Supplemental Fig. S1).

### CrMCA-II is a Ca^2+^- and redox-dependent arginine-specific protease

**Figure 1.**
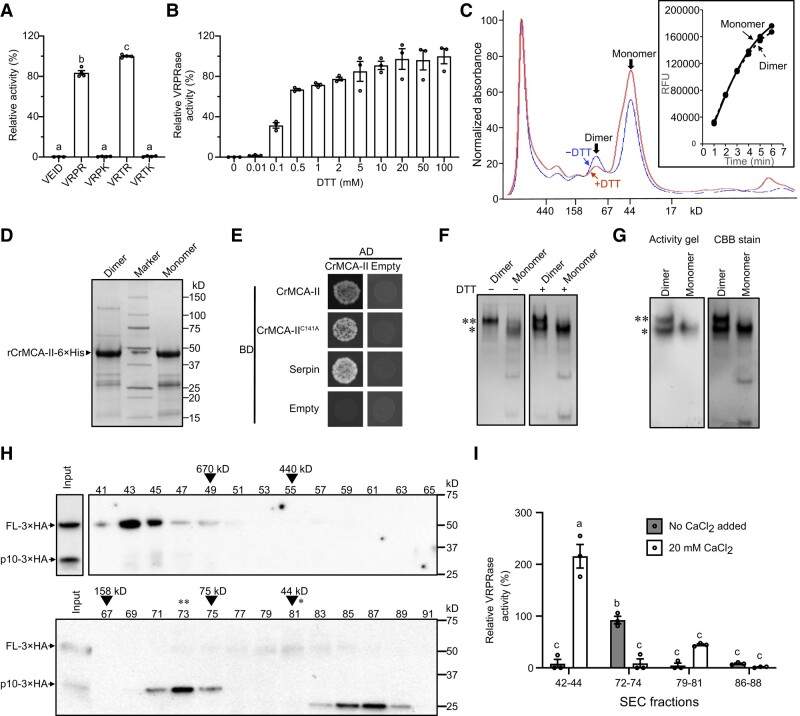
CrMCA-II is a redox-dependent arginine-specific protease prone to oligomerization. **A)** Proteolytic activity of rCrMCA-II against AMC-conjugated tetrapeptide substrates with Arg, Lys, or Asp at the P1 position, relative to Ac-VRTR-AMC, under optimal buffer conditions (50 mM Tris-HCl, pH 7.5, 25 mM NaCl, 20 mM CaCl_2_, 0.1% [w/v] CHAPS, and 7.5 mM DTT). **B)** Proteolytic activity of rCrMCA-II, as a function of DTT concentration, relative to 100 mM DTT, under optimal buffer conditions, with 50 *μ*M Ac-VRPR-AMC as a substrate. **C)** Size exclusion chromatography (SEC) analysis of rCrMCA-II in the presence or absence of 1 mM DTT. The insert shows proteolytic activity (relative fluorescence units, RFU) of monomeric and dimeric forms of rCrCMA-II separated in the absence of DTT against Ac-VRPR-AMC under optimal buffer conditions. **D)** SDS-PAGE analysis of dimer- and monomer-containing fractions from C separated without DTT. The gel was stained with Coomassie Brilliant Blue R250 (CBB). **E)** Yeast two-hybrid (Y2H) assay of CrMCA-II self-interaction and interaction between the intact (wild-type) protease and its catalytically inactive mutant CrMCA-II^C141A^. Interaction between CrMCA-II and serpin, an in vivo inhibitor of plant metacaspases, was used as a positive control. AD, GAL4 activation domain; BD, GAL4 DNA-binding domain. The results are a representative example of 2 independent experiments. **F)** Native PAGE of monomeric and dimeric forms of rCrMCA-II with (+) or without (−) DTT (7.5 mM). The gels were stained with CBB. **G)** In-gel proteolytic activity assay of monomeric and dimeric forms of CrMCA-II using native PAGE under optimal buffer conditions, with 50 *μ*M Ac-VRPR-AMC as a substrate. * and ** in **F)** and **G)** indicate positions of the monomer and dimer, respectively. **H)** Immunoblot analysis of total protein extracts isolated from the *Crmca-ii* mutant strain expressing wild-type *CrMCA-II* tagged with 3*×*HA (*Crmca-ii-23 CrMCA-II-3×HA*; see Fig. 3) and fractionated by SEC. Odd fractions from 41 to 91 were separated by SDS-PAGE and analyzed with an anti-HA antibody. The fractions containing protein standards with indicated molecular masses are marked with arrowheads. Based on the calibration curve, the predicted average molecular masses of proteins in fractions 43, 73, and 87 are 1,185 kD, 88.5 kD, and 26.4 kD, respectively. The theoretical masses of CrMCA-II-3*×*HA zymogen (full-length monomer, FL-3*×*HA) and C-terminal p10 fragment (p10–3*×*HA; generated via autocleavage after Arg-190) are 46.1 kD and 25.9 kD, respectively. The fractions enriched for CrMCA-II-3*×*HA monomers and dimers are denoted by * and **, respectively. **I)** Relative proteolytic activity of CrMCA-II-3*×*HA in a subset of SEC fractions from H enriched for megadalton assemblies (fractions 42 to 44), dimers (fractions 72 to 74), monomers (fractions 79 to 81), and a fragment smaller than p10 (fractions 86 to 88) against Ac-VRPR-AMC under optimal buffer conditions, with or without addition of 20 mM CaCl_2_. Note the presence of a basal level of cell-derived Ca^2+^ in the assay. The relative VRPRase activity in different SEC fractions was normalized by subtracting background activity caused by proteases other than CrMCA-II-3*×*HA present in the SEC fractions. This background activity was determined from measuring VRPRase activity in the corresponding SEC fractions obtained from *Crmca-ii* mutant strain expressing catalytically dead variant of CrMCA-II tagged with 3*×*HA (strain *Crmca-ii-23 CrMCA-II^C141A^-3×HA*; see Fig. 3 and Supplemental Fig. S7). Data in **A)**, **B)**, and **I)** represent the means ± standard error of the mean (Sem) of 3 **B and I**) or 4 **A)** measurements. Different letters indicate significant differences at *P* < 0.05, as determined by a 1-way **A)** or 2-way **I)** ANOVA with Tukey's honest significant difference test.

We purified bacterially produced recombinant His-tagged CrMCA-II (rCrMCA-II) using a HisTrap column followed by size exclusion chromatography (SEC). For reasons discussed later, the optimal buffer used for CrMCA-II proteolytic activity measurements and as a basis for further studies was 50 mM Tris pH 7.5, 25 mM NaCl, 20 mM CaCl_2_, 0.1% CHAPS, and 7.5 mM DTT. Similar to all previously studied MCAs, rCrMCA-II was unable to cleave after Asp (Fig. 1A). However, unlike other MCAs, rCrMCA-II displayed a strict preference for Arg over Lys at the P1 position in the tetrapeptide substrates, with only trace hydrolytic activity against Lys-containing substrates (Fig. 1A).

As with most MCAs, rCrMCA-II required millimolar concentrations of Ca^2+^ for activation (Supplemental Fig. S2A). An inhibitory effect of zinc on CrMCA-II (Supplemental Fig. S2B) was not as strong as that for MCA mcII-Pa from Norway spruce (*Picea abies*) (Bozhkov et al. 2005), but similar to human caspase 8 (Stennicke and Salvesan 1997). rCrMCA-II was active at neutral pH, with an optimum at pH 7.5 (Supplemental Fig. S2C), and was highly sensitive to arginal protease inhibitors (Supplemental Fig. S2D).

Notably, rCrMCA-II critically required a reducing agent for activation (Supplemental Fig. S2E), exhibiting dose-dependent activity with increasing concentration of DTT up to 20 mM (Fig. 1B). The predicted structure of the CrMCA-II catalytic center revealed Cys-329 in the vicinity of catalytic Cys-141 (5.2 Å, Supplemental Fig. S3), at a distance even closer than the distance between the catalytic dyad composed of Cys-141 and His-87 (6.5 Å), pointing to a possibility for a Cys–Cys bridge formation that may inhibit catalytic activity. However, the rCrMCA-II^C329A^ mutant was significantly less proteolytically active than the intact enzyme and still required reducing conditions for activity (Supplemental Fig. S2F). To explore this topic further, we examined 3 other Cys residues (Cys-11, Cys-23, and Cys-107) whose oxidation had been identified in previous redox proteome studies (Pérez-Pérez et al. 2017; McConnell et al. 2018). Notably, these 3 Cys residues are situated closely enough to potentially form Cys–Cys bridges (5.0 Å between Cys-11 and Cys-23, 3.2 Å between Cys-11 and Cys-107, Supplemental Fig. S3). Here we chose to introduce isosteric mutations of each Cys to Ser rather than Ala, in order to minimize possible structural perturbations. As with rCrMCA-II^C329A^, all 3 purified rCrMCA-II variants (C11S, C23S, and C107S) were active against the model substrate, but showed significantly lower catalytic activity compared to intact rCrMCA-II (∼20% to 75% of rCrMCA-II). Nevertheless, all variants still required a reducing agent for activation (Supplemental Fig. S2F).

In agreement with the results obtained for rCrMCA-II, addition of DTT to Chlamydomonas cell lysates prepared from the control (UVM4) strain also increased Arg-specific proteolytic activity, whereas hydrogen peroxide had the opposite effect (Supplemental Fig. S4, A to C). We were intrigued by the potential reciprocal regulation of CrMCA-II by DTT and H_2_O_2_. After activation by DTT, the subsequent introduction of H_2_O_2_ appeared to induce oxidative damage to the enzyme, resulting in lower activity even with re-addition of DTT (Supplemental Fig. S4D). This finding suggests that CrMCA-II relies on a reducing agent to uphold its active state but is also irreversibly sensitive to oxidative damage triggered by H_2_O_2_. Collectively, these results demonstrate that CrMCA-II is a Ca^2+^- and redox-dependent arginine-specific protease.

### CrMCA-II oligomerizes in vitro and in vivo

SEC profiles of rCrMCA-II in the buffer without DTT and Ca^2+^ displayed 2 peaks, corresponding to the monomeric (43.5 kD) and dimeric (87 kD) forms (Fig. 1C). When subjected to SDS-PAGE, both monomer- and dimer-containing SEC fractions exhibited full-length protein and autocleavage fragments of a similar molecular mass (Fig. 1D). We confirmed the dimerization by yeast two-hybrid (Y2H) assay (Fig. 1E). Caspases dimerize through the sixth β-sheet located in the p10 region (MacKenzie and Clark 2012). We attempted to identify the interaction region of CrMCA-II, but neither p20 nor p10 or linker regions could interact with full-length CrMCA-II in the Y2H assay (Supplemental Fig. S5), indicating that either the corresponding protein fragments are misfolded or the presence of 2 or even all 3 regions is required for dimerization.

The SEC fractions containing either monomeric or dimeric forms of rCrMCA-II were proteolytically active in buffer supplemented with DTT and Ca^2+^ (Fig. 1C, insert). As DTT may prevent protein–protein interaction by disrupting intermolecular disulfide bonds, the activity of the dimer-containing fraction may in fact be caused by monomers forming upon addition of DTT. Indeed, we observed a decreased dimer-to-monomer ratio when 1 mM DTT was added to the SEC buffer, confirming that DTT induces dimer-to-monomer transition (Fig. 1C). We further confirmed this notion using native gel electrophoresis (Fig. 1F). To unequivocally determine which of the 2 forms of rCrCMA-II represent the catalytically active protease, we performed an in-gel activity assay wherein monomeric and dimeric forms of rCrMCA-II were subjected to native electrophoresis followed by the addition of Ac-VRPR-AMC. Both monomers and dimers were able to cleave the fluorescent substrate (Fig. 1G), demonstrating that dimerization is dispensable for rCrMCA-II activation in vitro.

To investigate whether CrMCA-II dimerizes in vivo, we performed SEC of total protein extracts from a knockout Chlamydomonas strain complemented with a construct encoding HA-tagged wild-type CrMCA-II (*CrMCA-II-3×HA*; generation of transgenic strains is discussed later), followed by SDS-PAGE and immunoblot analysis of the SEC fractions with an anti-HA antibody (Fig. 1H). This analysis identified 3 major forms of CrMCA-II-3*×*HA co-existing in the cell extracts: monomer (peak fraction 81), dimer (peak fraction 73), and a megadalton-scale complex (peak fraction 43; Fig. 1H). While CrMCA-II-3*×*HA in the SEC fractions containing monomer and megadalton complex was present mainly as a zymogen (i.e. full-length inactive proenzyme), the dimer-containing fractions were represented by the autoprocessed, mature protease, generating a p10 fragment under denatured conditions that is recognized by the anti-HA antibody (Fig. 1H). This fragment results from the autocleavage at a conserved site (after Arg-190 in the case of CrMCA-II and based on sequence alignment with known cleavage sites from Arabidopsis type-II MCAs) within the linker region of type-II MCAs (Zhu et al. 2020; Supplemental Fig. S6). Besides 3 major forms of CrMCA-II-3*×*HA, the SEC analysis of the total protein extract has also revealed an abundance of a C-terminal fragment smaller than the p10 fragment present in a free, unbound form (peak fraction 87; Fig. 1H) and apparently representing a product of self-degradation (Watanabe and Lam 2011).

Since endogenous CrMCA-II exists as monomeric, dimeric, and large (megadalton-scale) species, we asked whether these species are proteolytically active in the substrate cleavage assay, at 2 contrasting levels of Ca^2+^, but otherwise maintaining optimal buffer conditions: (i) without adding any exogenous Ca^2+^, and (ii) with addition of 20 mM CaCl_2_. While the first conditions approximate the chemical environment in situ, with only a basal level of cell-derived Ca^2+^ present in the assay, the second conditions provide optimal levels of Ca^2+^ for detecting full activity of a proteolytically competent CrMCA-II. Ca^2+^ supplementation strongly enhanced VRPRase activity of the SEC fractions with megadalton and monomeric CrMCA-II species, largely represented by zymogen and exhibiting trace activities without adding extra Ca^2+^ (Fig. 1I). Conversely, the relatively high VRPRase activity of dimer-containing fractions represented by the autoprocessed mature enzyme was suppressed by extra Ca^2+^, presumably through self-degradation (Watanabe and Lam 2011). The SEC fractions containing a C-terminal fragment of CrMCA-II smaller than p10 displayed only trace activity, independently of the Ca^2+^ level (Fig. 1I). It is important to note that the proteolytic activities shown in Fig. 1I were normalized by subtracting background VRPRase activities detected in the corresponding SEC fractions due to the presence of other proteases with substrate specificity similar to that of CrMCA-II. These background activities were determined by analyzing SEC fractions from the same knockout strain that harbors a construct encoding a catalytically dead variant of CrMCA-II (*CrMCA-II^C141A^-3×HA*; Supplemental Fig. S7).

Results of SEC, immunoblotting and proteolytic activity assays together have 2 important implications. First, the pattern of the CrMCA-II protein in SDS-PAGE of Chlamydomonas cell lysates is indicative of its proteolytic competence. Second, it is highly likely that it is a dimer representing a proteolytically active form of CrMCA-II in vivo, whereas monomeric and megadalton CrMCA-II species are present as catalytically competent but inactive zymogen molecules.

### Development of CrMCA-II-specific chemical probes

To determine the substrate specificity of CrMCA-II beyond P1 position and to develop optimal substrates, we employed hybrid combinatorial substrate library (HyCoSuL) screening combining both natural and unnatural amino acids (Poreba et al. 2017). Using the library with fixed Arg at the P1 position, we established the enzyme preferences at the P2, P3, and P4 positions (Supplemental Fig. S8A). The screening revealed a broad substrate specificity for rCrMCA-II toward amino acids in all 3 positions. Of note, the temperature in the physiologically relevant range of 24 to 42 °C had no effect on the P4–P2 substrate preferences of rCrMCA-II (Supplemental Fig. S8B).

**Figure 2.**
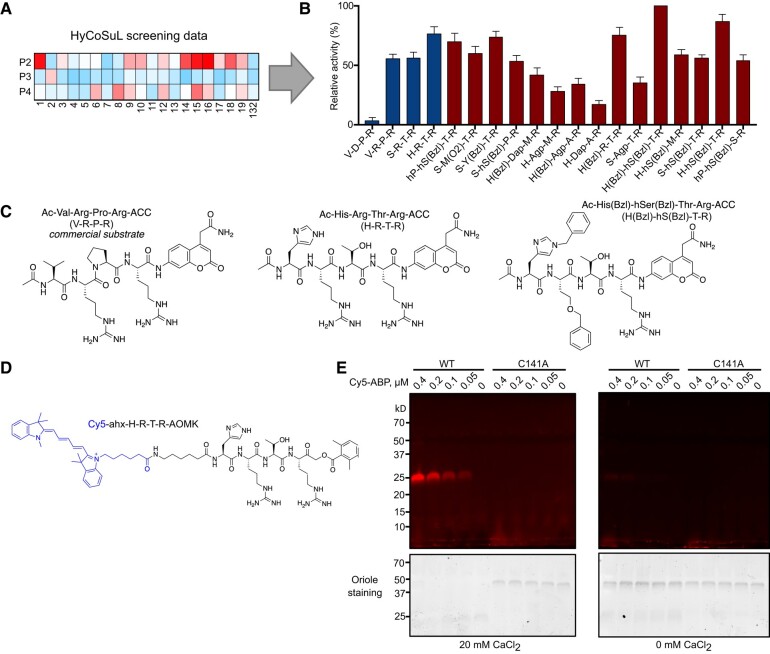
A chemical toolbox for CrMCA-II. **A)** HyCoSuL screening data were used to extract optimal peptide sequences for CrMCA-II substrates and activity-based probes. **B)** Proteolytic activity of recombinant CrMCA-II (rCrMCA-II) against individual fluorescent substrates containing natural (blue bars) or unnatural (red bars) amino acids. Data represent the means ± standard error of the mean (Sem) of triplicate measurements. **C)** Structures of rCrMCA-II substrates: Ac-VRPR-ACC (commercially available as Ac-VRPR-AMC), Ac-HRTR-ACC (most preferred with natural amino acids), and Ac-H(Bzl)-hS(Bzl)-TR-ACC (most preferred with unnatural amino acids). **D)** Structure of Cy5-labeled covalent activity-based probe (Cy5-ABP) with an HRTR peptide motif and AOMK electrophilic warhead. **E)** Binding of different concentrations of Cy5-ABP to 0.4 *μ*M wild-type (WT) rCrMCA-II or its catalytically inactive variant (C141A) under optimal buffer conditions, in the absence or presence of 20 mM CaCl_2_, as visualized after SDS-PAGE. Oriole staining served as a loading control.

To gain insight into enzyme preferences, we synthesized a set of individual tetrapeptide substrates bearing a 7-amino-4-carbamoylmethylcoumarin (ACC) fluorophore reporter, which we subjected to rCrMCA-II hydrolysis (Fig. 2, A to C; Supplemental Fig. S9; Supplemental Data Set 1). This screening enabled us to identify 2 substrates, 1 composed of natural amino acids, Ac-His-Arg-Thr-Arg-ACC (Ac-HRTR-ACC), and 1 with unnatural amino acids, Ac-His(Bzl)-hSer(Bzl)-Thr-Arg-ACC (Ac- H(Bzl)-hS(Bzl)-TR-ACC), both of which showed a high cleavage rate by rCrMCA-II. The catalytic activity of rCrMCA-II on these substrates was significantly higher than that on the commercially available and commonly used MCA and paracaspase substrate Ac-VRPR-ACC (Table 1, Fig. 2, B and C), which was designed from the optimal substrate preference of Arabidopsis AtMCA-IIf (Vercammen et al. 2006), making them efficient probes for measuring CrMCA-II activity.

**Table 1.** Catalytic activity of rCrMCA-II against 3 selected substrates

Substrate	*K_M_* (*μ*M)	*k*_cat_ (s^−1^)	*k*_cat_/*K_M_* (M^−1^ s^−1^)
Ac-Val-Arg-Pro-Arg-ACC	56.5	10.3	1.82 × 10^5^
Ac-His-Arg-Thr-Arg-ACC	48.1	15.9	3.31 × 10^5^
Ac-His(Bzl)-hSer(Bzl)-Thr-Arg-ACC	31.0	13.0	4.20 × 10^5^

A considerable benefit of analyzing protease substrate preferences using HyCoSuL technology is the development of a peptide that perfectly fits into the active site of the investigated enzymes. Therefore, we used the rCrMCA-II preferred peptidic substrates to design fluorescently labeled activity-based probes (ABPs), that is covalent inhibitors that bind to the enzyme active site in an irreversible manner. To this end, we labeled HRTR and H(Bzl)-hS(Bzl)-TR peptides with a cyanine 5 (Cy5) tag through an 6-aminohexanoic acid (Ahx) linker to separate the fluorophore and peptide. We selected the acyloxymethyl ketone group (AOMK) to use as the warhead (Fig. 2D), as this electrophile is known to covalently react with the catalytic Cys residue of Cys proteases (Kato et al. 2005). We thus developed 2 CrMCA-II ABPs, Cy5-ahx-HRTR-AOMK and Cy5-ahx-H(Bzl)-hS(Bzl)-TR-AOMK (Fig. 2D, Supplemental Fig. S10, and Supplemental Table S1).

To determine whether the ABPs can differentiate between intact wild-type (WT) and the catalytically dead mutant of CrMCA-II, as well as between mature WT CrMCA-II and its zymogen, we incubated purified rCrMCA-II or rCrMCA-II^C141A^ with Cy5-ahx-HRTR-AOMK under optimal buffer conditions in the presence or absence of Ca^2+^ followed by SDS-PAGE. As shown in Fig. 2E, we observed no labeling for rCrMCA-II^C141A^ regardless of the presence of Ca^2+^, whereas the ABP labeled only the autoprocessed, mature form of rCrMCA-II generated in the presence of Ca^2+^ in a dose-dependent manner. This result indicates that the ABP can distinguish between active rCrMCA-II and its zymogen. The fragment of mature rCrMCA-II covalently bound by the Cy5 probe is expected to include the p20 region as well as a part of the linker to Arg-190, migrating as a band of ∼25 kD (Fig. 2E). These results demonstrate that our Cy5-labeled ABPs are useful tools for detecting mature CrMCA-II enzyme.

### Generation of *CrMCA-II* knockout mutants and complementation strains

**Figure 3.**
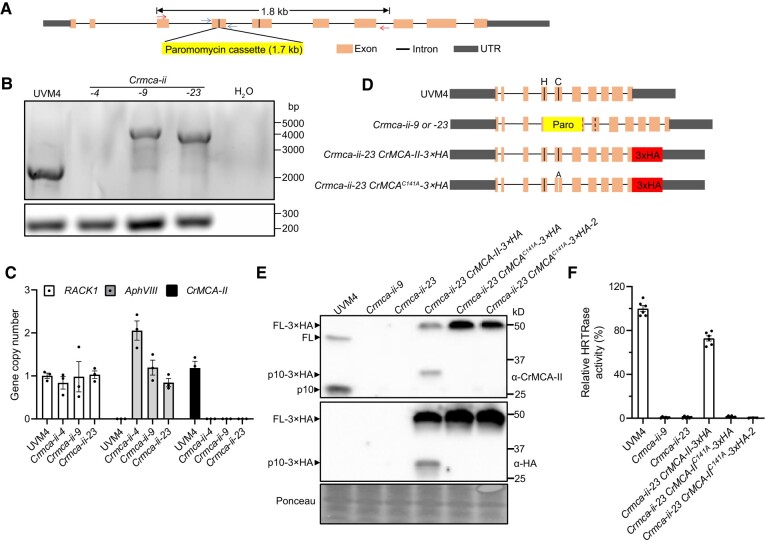
Generation of the *Crmca-ii* mutant and complementation strains in the UVM4 background. **A)** Diagram of the editing site of *CrMCA-II*. Red and blue arrows depict pairs of primers used for PCR and quantitative PCR (qPCR) analyses, respectively. The vertical lines in exons 4 and 5 indicate the position of the catalytic dyad of His-87 and Cys-141. **B)** PCR test using the primer pair depicted in **A)** with genomic DNA as a template. Distilled water was used as a negative control. Bottom panel shows PCR product for *RACK1* (positive control). *Crmca-ii-4*, *Crmca-ii-9*, and *Crmca-ii-23* are presumed mutant strains. The agarose gel was stained with GelRed. **C)** qPCR analysis of *RACK1*, *AphvIII*, and *CrMCA-II* copy number using the primer pair depicted in **A)** and genomic DNA as a template to evaluate genome insertion events. Data represent the means ± standard error of the mean (Sem) of triplicate measurements. Paro, paromomycin resistance cassette. **D)** Diagram of the *CrMCA-II* gene structure in the generated strains. The vertical lines indicate the position of the catalytic dyad of His-87 (H) and Cys-141 (C); A represents the Cys-141 for Ala (A) substitution in strains transformed with a catalytically inactive mutant. The dashed vertical lines corresponding to the position of the catalytic dyad indicate abolished transcription of the *CrMCA-II* gene in the mutant strains. **E)** Immunoblot analysis of CrMCA-II in UVM4, *CrMCA-II* mutant (*Crmca-ii-9* and *Crmca-ii-23*), and complementation strains (1 *Crmca-ii-23 CrMCA-II-3×HA* strain and 2 *Crmca-ii-23 CrMCA-II^C141A^-3×HA* strains [independent colonies from the same transformation event]) using anti-CrMCA-II and anti-HA antibodies. Ponceau staining of the PVDF membrane was used as a loading control. **F)** Peptide (Ac-HRTR-ACC) cleavage assay of cell lysates (total protein extracts) from the same strains as in **E)**. Data represent the means ± Sem from 2 independent biological experiments, each including 3 replicate measurements.

To study the physiological roles of CrMCA-II, we generated *Crmca-ii* mutant strains using the recently established targeted insertional mutagenesis method based on the clustered regularly interspaced short palindromic repeat (CRISPR)/CRISPR-associated nuclease 9 (Cas9) system (Picariello et al. 2020). We obtained 3 strains (*Crmca-ii-4*, *Crmca-ii-9*, and *Crmca-ii-23*) lacking *CrMCA-II* expression. PCR amplification of genomic DNA from strains *Crmca-ii-9* and *Crmca-ii-23* with a pair of primers flanking the insertion site resulted in a product of around 3.5 kb instead of 1.8 kb in the control strain (Fig. 3, A and B; All primers used in this study are provided in Supplemental Data Set 2). Sequencing confirmed the insertion of the *AphVIII* cassette conferring paromomycin resistance in the genomic DNA (Supplemental Fig. S11). A third mutant, *Crmca-ii-4*, produced no amplicon after colony PCR (Fig. 3B), suggesting a larger insertion.

We used quantitative PCR (qPCR) for estimating the number of gene copies and insertion events in the genome as an alternative to a more time-consuming Southern blot analysis (Hoebeeck et al. 2007). While the *Crmca-ii-9* and *Crmca-ii-23* strains contained a single *AphVIII* gene copy, confirming single insertion events, *Crmca-ii-4* presumably had 2 *AphVIII* gene copies inserted into the genome (Fig. 3, C and D). Furthermore, qPCR analysis confirmed that none of the 3 *Crmca-ii* mutant strains contain an intact *CrMCA-II* gene (Fig. 3C).

To verify the lack of CrMCA-II protein in the generated mutants, we used an antibody raised against a 16-amino acid–long peptide from the p10 region of CrMCA-II (Supplemental Fig. S12A). The antibody recognized both the zymogen (∼42.4 kD) and a fragment composed of p10 and a part of the linker region generated via autocleavage (migrating as ∼25 kD but estimated to be 22 kD) on immunoblots of total protein extracts isolated from the control strain (UVM4; Fig. 3E and Supplemental Fig. S12B). By contrast, none of the 3 *Crmca-ii* strains revealed the presence of CrMCA-II protein (Fig. 3E and Supplemental Fig. S12B), indicating that all 3 strains are knockouts. We also analyzed cell lysates isolated from the control strain and knockouts for proteolytic activity of CrMCA-II using the optimized substrate Ac-HRTR-ACC. To minimize the risk that cleavage of this substrate by other Chlamydomonas proteases could mask the effect of CrMCA-II deficiency, we blocked activity of most aspartate, cysteine and serine proteases using a combination of aprotinin, pepstatin A, PMSF, and Pefabloc SC. While we readily detected HRTRase activity in the lysates from UVM4 strain, we barely detected any in the lysates from the *Crmca-ii* strains (Fig. 3F and Supplemental Fig. S12C), making them a robust tool for functional studies.

To further expand our toolbox, we cloned the native *CrMCA-II* promoter and genomic DNA of *CrMCA-II* into the MoClo system (Crozet et al. 2018), and obtained mutant strains harboring a construct encoding HA-tagged WT CrMCA-II or catalytically dead CrMCA-II^C141A^ (Fig. 3D). While complementation with the WT construct produced a mature, catalytically active protease, complementation with the autoprocessing-deficient variant *CrMCA-II^C141A^* failed to restore HRTRase activity in cell lysates (Fig. 3, E and F).

### CrMCA-II plays a cytoprotective role under HS independently of its protease activity

**Figure 4.**
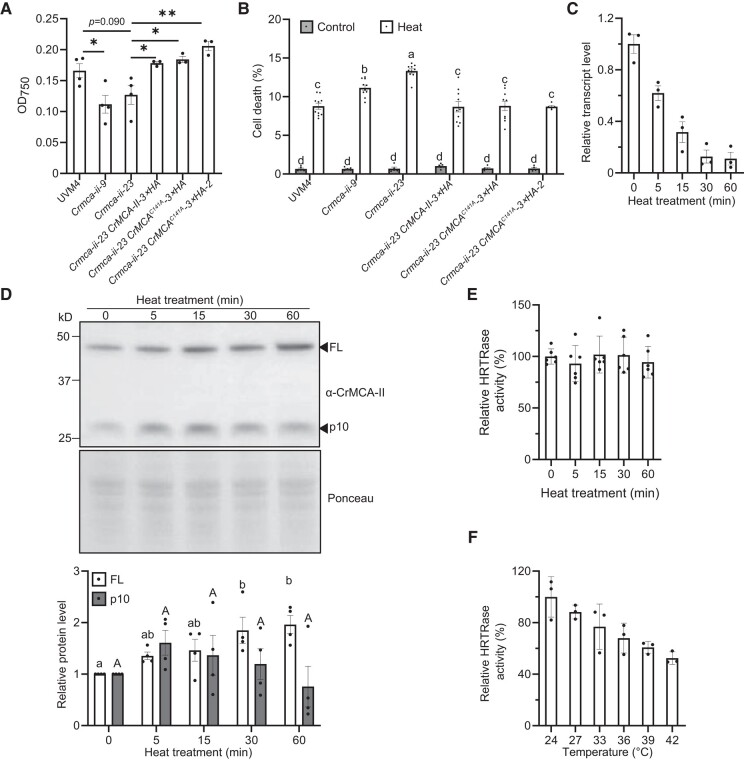
CrMCA-II plays a cytoprotective role during HS. **A)** Growth of UVM4, *Crmca-ii* mutants, and complemented strains after HS, as estimated by measuring optical density of liquid cultures at 750 nm (OD750) 6 d after recovery from 2 h of HS at 42 °C. One hundred *μ*L cells at log phase (5 to 6 × 10^6^ cells mL^−1^) were heat-treated for 2 h at 42 °C. Fifty *μ*L culture was then inoculated into 3 mL fresh TAP medium for recovery and optical density measurements at 750 nm (OD750). Data represent the means ± Sem of 1 representative example of 3 independent experiments, each including 3 or 4 measurements. **P < 0.01, *P < 0.05, as determined by a *t*-test. **B)** Percentage of cell death in the UVM4, *Crmca-ii* mutants, and complemented strains growing under control conditions (23 °C; control) or subjected to 60 min of HS at 42 °C (Heat), as determined by fluorescein diacetate (FDA) staining. Data represent the means ± standard error of the mean (Sem) of 5 independent experiments. Each experiment included 1 or 2 measurements, and each measurement included 600 to 900 cell counts per strain and condition. Different letters indicate significant differences at *P* < 0.05, as determined by a 2-way ANOVA with Tukey's honest significant difference test. **C)** Relative *CrMCA-II* transcript levels in the UVM4 strain at 0, 5, 15, 30, and 60 min into HS at 42 °C, as determined by RT-qPCR. The transcript level at 0 min was set to 1. Data represent the means ± Sem of 3 independent biological experiments. **D)** Immunoblot analysis of full-length (FL) and the p10 fragment of CrMCA-II in total protein extracts isolated from the UVM4 strain at 0, 5, 15, 30, and 60 min of HS at 42 °C. Ponceau staining of the PVDF membrane was used as a loading control. Data on chart represent means ± Sem of relative abundance of FL CrMCA-II and p10 based on densitometry analysis of the corresponding bands in 4 independent biological experiments. The levels of FL and p10 at 0 min were set to 1. Different letters indicate significant differences at *P* < 0.05, as determined by a 2-way ANOVA with Tukey's honest significant difference test. **E)** Relative HRTRase activity of cell lysates isolated from the UVM4 strain at 0, 5, 15, 30, and 60 min into HS at 42 °C. Data represent the means ± Sem of 2 independent biological experiments, each including triplicate measurements. **F)** Relative HRTRase activity of rCrMCA-II pretreated for 5 min at different temperatures in Ca^2+^- and DTT-free buffer (nonpermissive conditions) before activity measurements under optimal buffer conditions. Data represent the means ± Sem of triplicate measurements.

We took advantage of the generated probes and algal strains to explore the role of CrMCA-II in vivo. We compared colony growth of UVM4, knockouts, and complementation strains under a wide range of nutrient and environmental conditions, but did not observe any difference in their growth phenotypes (Supplemental Fig. S13). Since no cells survived in the samples treated with HS at 42 °C for 2 h after 20 d of incubation on plates, we modified the test by growing HS-treated cells in liquid rather than on solidified Tris-acetate phosphate (TAP) medium. We observed a significant difference in algal growth following HS, with *Crmca-ii* strains exhibiting suppressed growth compared to UVM4 (Fig. 4A and Supplemental Fig. S14). The complemented strains with WT *CrMCA-II* restored cell growth. Interestingly, complementation with *CrMCA-II^C141A^* in 2 independent strains did so too (Fig. 4A and Supplemental Fig. S14). We then compared the frequency of cell death in the algal strains after a 60 min HS at 42 °C using fluorescein diacetate (FDA) staining (Fig. 4B). Consistent with the results of cell growth analysis, the mutant strains had a higher frequency of cell death than the UVM4 strain, whereas complementation with both WT CrMCA-II and CrMCA-II^C141A^ restored cell viability (Fig. 4B). Taken together, these data demonstrate that CrMCA-II sustains cell growth and viability of Chlamydomonas under HS, and that this cytoprotective effect of CrMCA-II is independent of its protease activity.

Next, we wondered what happened to CrMCA-II during 42 °C HS at the transcriptional and protein levels. We observed that while HS leads to a steady decrease of *CrMCA-II* transcript levels in the UVM4 strain (Fig. 4C), the same HS treatment promoted zymogen accumulation without significantly altering the abundance of the mature form of the enzyme, detected through the formation of p10 fragment recognized by the anti-CrMCA-II antibody (Fig. 4D). In agreement, HRTRase activity of cell lysates remained constant over the 60 min duration of HS (Fig. 4E). These data indicated that HS triggers a trans-acting mechanism and/or conformational changes suppressing CrMCA-II activation and favoring zymogen accumulation. To clarify this further, we measured HRTRase activity of purified rCrMCA-II pretreated for 5 min in Ca^2+^- and DTT-free buffer (nonpermissive conditions) at various temperatures ranging from 24 °C to 42 °C and observed a decrease in protease activity with higher pretreatment temperature following addition of Ca^2+^ and DTT (Fig. 4F). We thus assumed that heat-induced suppression of CrMCA-II activation may be due to conformational changes of the zymogen.

### CrMCA-II is associated with the PM but translocates to the cytoplasm during HS

**Figure 5.**
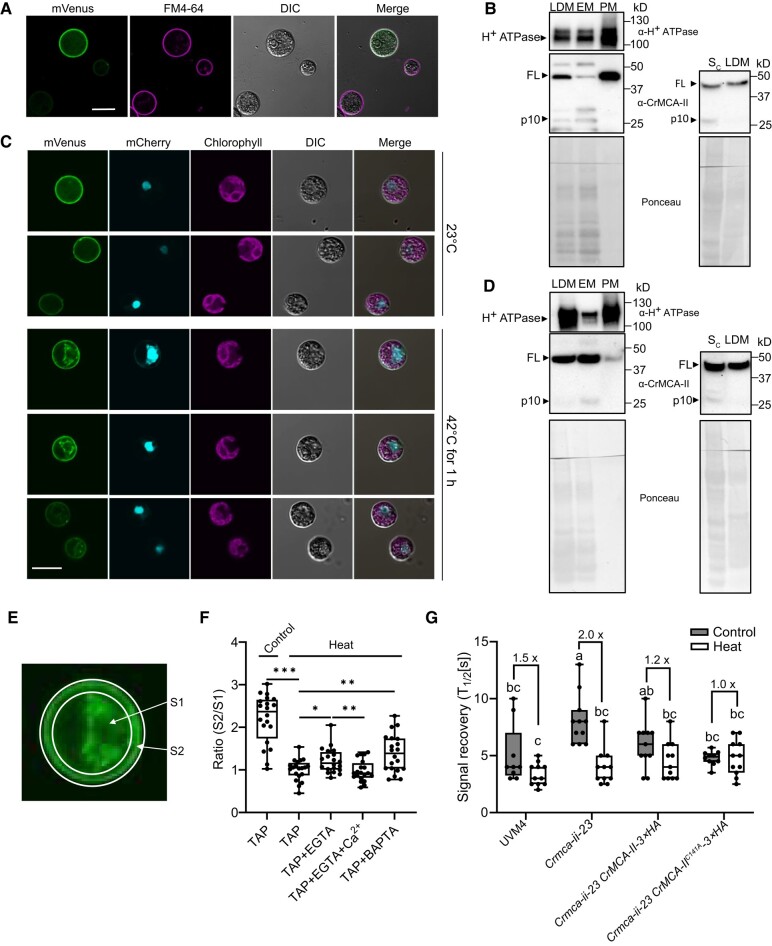
CrMCA-II localizes to the plasma membrane and modulates its fluidity. **A)** Plasma membrane (PM) localization of CrMCA-II-mVenus (green) in unstressed UVM4 cells (grown at 23 °C). Cells were stained with FM4-64 (magenta). Scale bar, 10 *μ*m. **B)** Immunoblot analysis of H^+^ ATPase and CrMCA-II in different subcellular fractions prepared from unstressed UVM4 cells. LDM, low-density membrane fraction; PM, plasma membrane fraction; EM, endomembrane fraction; S_c_, concentrated soluble proteins. Ponceau staining of the PVDF membranes were used as loading controls. FL, full-length CrMCA-II. **C)** PM-to-cytoplasm translocation of CrMCA-II-mVenus (green) after HS at 42 °C for 60 min. Cells co-expressed mCherry-NLS (turquoise). Scale bar, 10 *μ*m. **D)** Immunoblot analysis of different subcellular fractions prepared as in **B)**, but after HS at 42 °C for 60 min. **E)** Diagram showing the separation of the whole cell area into the cytoplasm area (S1) and PM area (S2) for CrMCA-II-mVenus PM-to-cytoplasm signal ratio quantification in **F)**. **F)** PM-to-cytoplasm ratio of CrMCA-II-mVenus in unstressed (23 °C, control) and heat-stressed (42 °C, heat) cells grown in TAP medium (0.34 mM CaCl_2_) with or without EGTA (0.75 mM), BAPTA (0.5 mM), or presence of both EGTA (0.75 mM) and CaCl_2_ (1 mM). ***P < 0.001; **P < 0.01, *P < 0.05, as determined by a *t*-test. **G)** Recovery rate of DiOC6(3) fluorescence signal following photobleaching (FRAP) of cells from the indicated strains grown under unstressed conditions (23 °C, control) or HS (39 °C for 60 min, heat). Numbers on the top of the chart indicate fold-increase of PM fluidity in heat-stressed compared to unstressed cells. Different letters indicate significant differences at *P* < 0.05, as determined by a 2-way ANOVA with Tukey's honest significant difference test. In **F)** and **G)**, the upper and lower box boundaries respectively represent the first and third quantiles, horizontal lines denote the median, and whiskers indicate the highest and lowest values. Data in **F)** and **G)** are from 3 independent biological experiments, each including at least 3 measurements (individual cells).

Given the thermoprotective role of CrMCA-II, we wondered where the enzyme was located in a Chlamydomonas cell before and after onset of HS. To study the subcellular localization of CrMCA-II, we assembled an expression vector with a constitutive *PSAD* promoter, a genomic DNA fragment of *CrMCA-II*, *mVenus*, and the *PSAD* terminator. After introduction into UVM4 cells (Neupert et al. 2009), we obtained a strain producing CrMCA-II with a C-terminal mVenus tag, named *CrMCA-II*-overexpressor 14#3 (OE14 #3). Confocal microscopy analysis of unstressed OE14 #3 cells showed a strong mVenus signal co-localizing with the PM stained with the dye FM4-64 (Fig. 5A). We confirmed the PM localization of CrMCA-II-mVenus in strain CC-4533, which unlike UVM4 has an intact cell wall and flagella (Supplemental Fig. S15, Zhang et al. 2022a, b), suggesting that this localization is a strain-independent characteristic of Chlamydomonas.

To provide independent biochemical evidence for the PM localization of CrMCA-II, we subjected proteins from several fractions including cytosolic, low-density membrane (LDM, including both PM and endomembranes [EM]), and separated PM and EM (Supplemental Fig. S16) to immunoblotting with antibodies against CrMCA-II and H^+^ ATPase, a marker for the PM (Norling et al. 1996). We observed an enrichment for H^+^ ATPase in the PM fraction, which also displayed a strong accumulation for CrMCA-II, in agreement with the microscopy data (Fig. 5B, left blot). Although confocal microscopy failed to detect cytoplasmic CrMCA-II-mVenus signal in unstressed cells (Fig. 5A), the concentrated soluble protein fraction (precipitated and dissolved in the same volume as LDM fraction) exhibited a similar level of CrMCA-II as the LDM fraction (Fig. 5B, right blot). Interestingly, CrMCA-II in the PM fraction was present only as a zymogen, in contrast to the cytosolic and EM fractions containing both zymogen and mature enzyme (represented by the p10 fragment; Fig. 5B). Taken together, these results confirm the PM localization of CrMCA-II in unstressed cells, but also point to the existence of a cytoplasmic pool for this protein. In addition, the data suggest that while the PM pool of CrMCA-II is represented exclusively by the zymogen, the cytoplasmic pool is partitioned between zymogen and mature protease.

We studied the localization of CrMCA-II under HS using a strain accumulating the fluorescent protein mCherry fused to a simian virus 40 nuclear localization signal (SV40), in the OE14 #3 background (Fig. 5C). When cells were exposed to HS at 42 °C for 60 min, we observed the appearance of a strong mVenus signal in the perinuclear cytoplasm (Fig. 5C). Subcellular fractionation followed by immunoblotting corroborated the relocation of CrMCA-II to the cytoplasm, as the abundance of the protein significantly increased in both soluble and EM fractions (Fig. 5D). Thus, the ratio of PM to cytoplasmic fluorescent signals of CrMCA-II decreased by more than 2 times in the HS-treated compared to unstressed cells (Fig. 5, E and F). Importantly, proteolytic activity of CrMCA-II was not required for its PM-to-cytoplasm re-distribution, as revealed by the microscopy analysis of a strain accumulating mVenus tagged CrMCA-II^C141A^ (Supplemental Fig. S17, A and B).

Next, to determine whether the HS-induced accumulation of cytoplasmic CrMCA-II is a consequence of its translocation from the PM or de novo protein synthesis, we treated cells with cycloheximide (CHX), an inhibitor of translation (Schneider-Poetsch et al. 2010), prior to HS. Since CHX did not prevent or alleviate perinuclear signal (Supplemental Fig. S17C), we conclude that the enhanced cytoplasmic accumulation of CrMCA-II during HS is a result of PM-to-cytoplasm translocation rather than de novo protein synthesis.

HS is known to induce the influx of Ca^2+^ through the PM (Saidi et al. 2009). MCAs generally contain 2 Ca^2+^ binding sites, 1 with high affinity and unknown function and another site with low affinity that is involved in catalytic activation (Klemenčič and Funk 2019). During HS, Ca^2+^ influx results in an increase of intracellular Ca^2+^ concentration to a micromolar level (summarized in Schroda et al. 2015), i.e. to the range where MCAs bind Ca^2+^ with high affinity. To test whether the influx of Ca^2+^ through the PM is involved in the PM-to-cytoplasm translocation of CrMCA-II, we pretreated OE14#3 cells in log phase with 0.75 mM of the Ca^2+^ chelators EGTA or 1,2-bis(o-aminophenoxy) ethane-N,N,N,N′-tetraacetic acid (BAPTA) prior to HS. Calcium chelation led to a moderate but statistically significant suppression of CrMCA-II translocation, whereas addition of excess Ca^2+^ to the EGTA-treated cells fully reverted this effect (Fig. 5F), suggesting that Ca^2+^ influx during HS does play a role in the PM-to-cytoplasm translocation of CrMCA-II.

### CrMCA-II modulates PM fluidity

We hypothesized that the molecular mechanism underlying the cytoprotective effect of CrMCA-II under HS might be related to its potential role in modulating PM fluidity, a dynamic property of PM critically involved in HS signaling (Saidi et al. 2009; Niu and Xiang 2018) and activation of cell death (Tekpli et al. 2013). To test this hypothesis, we stained the algal strains with DiOC6(3), a lipophilic green fluorescent dye with a high affinity for the Chlamydomonas PM (Long et al. 2016), followed by a fluorescence recovery after photobleaching (FRAP) assay. An attempt of running the assay at 42 °C failed due to hypersensitivity of the heat-treated cells to PM photobleaching that resulted in cell collapse, so we dropped the HS temperature to 39 °C.

We did not observe any significant differences in the proportion of the initial signal recovery of DiOC6(3) fluorescence at the PM between different strains and regardless of the temperature (Supplemental Fig. S18). However, CrMCA-II deficiency led to a strong decrease of signal recovery rate (T_1/2_ increase) of DiOC6(3) in unstressed cells, compared to UVM4 and both WT and *CrMCA-II^C141A^* complementation strains (Fig. 5G). While UVM4 and complementation strains generally responded to HS by a mild increase in their PM fluidity, this increase was significantly augmented in the CrMCA-II deficient mutant (Fig. 5G, Supplemental Fig. S19). These results indicate that CrMCA-II controls PM fluidity by preventing PM rigidization in unstressed conditions, which also avoids hyperfluidization in response to HS. Similar to cytoprotection and PM localization, this function of CrMCA-II occurs independently of its protease activity.

## Discussion

### Oligomerization of CrMCA-II

The absence of oligomerization-dependent activation of MCAs has been proposed as a generic feature distinguishing them from caspases and paracaspases (McLuskey and Mottram 2015; Minina et al. 2020). Our observations with Chlamydomonas CrMCA-II are not fully consistent with this notion. First, bacterially produced CrMCA-II can form monomers and dimers, which are both catalytically active (Fig. 1, C, D, and G). While dimerization in this case may be due to a high concentration of the recombinant protein, the results of Y2H assay do not support this assumption (Fig. 1E). Second, Chlamydomonas protein extracts contain monomeric, dimeric, and megadalton-size CrMCA-II species, of which only dimers appear to be fully active (Fig. 1, H and I).

Our observation that dimerization of endogenous CrMCA-II correlates with the autocleavage yielding the p10 fragment (Fig. 1H) is in agreement with a hypothetical model for type-II MCA maturation, which postulated that autocleaved MCA molecules form a catalytically active homodimer (Lam and Zhang 2012). One cannot completely rule out the possibility that protein species from Chlamydomonas cell extracts with a molecular mass corresponding to the CrMCA-II dimer and recognized by the specific antibody are in fact heterodimers composed of CrMCA-II and another protein of a similar molecular mass, although the likelihood of such scenario is low.

The structural details of CrMCA-II dimerization remain unknown. While type-II MCAs have a similar β-strand arrangement as caspases, the L2 loop is embedded in the potential dimerization interface, making it impossible for the dimerization to occur in the same way as in caspases (Zhu et al. 2020). However, unlike caspases, type-II MCAs have a long, intrinsically disordered linker between the p20 and p10 regions (Supplemental Fig. S3), providing an alternative interface for dimerization. Although we failed to detect interaction between the individually produced linker and the full-length CrMCA-II in the Y2H assay (Supplemental Fig. S5), this negative result may be due to large conformational changes of the disordered linker in yeast. Structural data for catalytically mature CrMCA-II are required to unambiguously determine which structural elements serve as the dimerization interface, as well as the molecular topology of the dimer. Likewise, structure–function understanding of the megadalton-scale, CrMCA-II zymogen-containing complex identified in Chlamydomonas extracts awaits further studies.

### CrMCA-II is a redox-dependent protease

Reducing conditions are crucial for CrMCA-II activation both in vitro (Supplemental Fig. S2E) and in cell lysates (Supplemental Fig. S4, A and B), and at the same time stimulate dimer-to-monomer transition of rCrMCA-II (Fig. 1F), suggesting that Cys–Cys disulfide bond(s) might contribute to CrMCA-II dimerization. The redox-regulated activity of a type-III MCA from the marine diatom *Phaeodactylum tricornutum* was previously shown to correlate with the formation of a potential disulfide bond between Cys-202 and Cys-259 (Graff van Creveld et al. 2021). While these Cys residues are not conserved in CrMCA-II, we reasoned that the formation of a disulfide bond between catalytic Cys-141 and the closely located Cys-329 would explain the strict requirement of reducing conditions for the protease activity of CrMCA-II (Supplemental Fig. S3). However, the C329A mutation did not render protease activity independent of the reducing agent. Furthermore, we mutated Cys-11, Cys-23, and Cys-107, which were identified as oxidized in published redox thiol proteome data sets of Chlamydomonas (Pérez-Pérez et al. 2017; McConnell et al. 2018), and discovered that these CrMCA-II variants also depend on a reducing agent for their activity (Supplemental Fig. S2F). These results lead to 2 equally plausible scenarios: (i) An oxidized Cys residue blocking enzyme activity is not among the Cys residues tested here by substituting for Ala or Ser; and (ii) there is more than 1 Cys residue subjected to oxidation that blocks activity.

An important finding is that all 4 rCrMCA-II Cys mutants (C11S, C23S, C107S, and C329A) are catalytically active under optimal buffer conditions, but show significantly decreased activity. This result indicates that, although not critical, there is positive selection for Cys at these positions. Presumably they need to be maintained in their reduced free Cys form for optimal catalytic activity, providing the potential for redox regulation. However, to which extent these reduced Cys are actually used for regulation of CrMCA-II enzyme activity depending on intracellular redox potential has yet to be investigated, calling for further research to uncover an underlying mechanism and its physiological implications in vivo.

For type-II MCAs in Arabidopsis, results point toward a potential for redox regulation for at least 2 enzymes. In the Ca^2+^-independent AtMCA-IIf, oxidation of the catalytic Cys by S-nitrosylation has been shown, and is hypothesized to play a central role for regulation of the proteolytic activity (Belenghi et al 2007). In a recent large-scale quantification of Cys oxidation in the Arabidopsis proteome, Ca^2+^-dependent AtMCA-IIa was identified as being approximately 20% oxidized at the catalytic Cys, possibly due to S-nitrosylation (Huang et al 2023). In the case of Chlamydomonas CrMCA-II, its redox dependence might be a regulatory mechanism related to localization to the PM, a major site for reactive oxygen species (ROS) production, perception and signal transduction in the cell (Nordzieke and Medraño-Fernandez 2018), and responsible for thermotolerance and other physiological functions of this MCA yet to be identified.

### Substrate specificity of CrMCA-II

Without regard to the non-proteolytic role of CrMCA-II in thermotolerance, the bacterially produced enzyme exhibits a multifold higher preference for Arg than for Lys at the P1 position in tetrapeptide substrates (Fig. 1A), suggesting that the S1 binding pocket of CrMCA-II has a higher affinity for Arg than for Lys (Stael et al. 2023). Whether this result holds true in vivo is unknown and necessitates information about cleavage sites in the native targets. In fact, a strong preference for Arg over Lys in peptidic substrates is a common feature of MCAs (Minina et al. 2017), which is however not obvious when analyzing cleavage sites of the target proteins (Sundström et al. 2009; Tsiatsiani et al. 2013). Thus, the residues at P1′ to P4′ of the substrates may also affect the binding and activity of MCAs during cleavage.

As for a specificity beyond P1, CrMCA-II displayed a clear preference for a basic amino acid at the P3 site (Supplemental Fig. S8), similar to Arabidopsis AtMCA-IIf (Vercammen et al. 2006). Accordingly, substitution of Arg for Asp at P3 of the commercially available VRPR-based substrate greatly diminished proteolytic activity of CrMCA-II (Fig. 2B). However, unlike AtMCA-IIf, which prefers Pro, Tyr, or Phe at P2 and branched chain Ile or Val at the P4 site (Vercammen et al. 2006), CrMCA-II instead favors substrates with Ala, Thr, or Ser at P2 and His or Ser at P4 (Supplemental Fig. S8). This comparison suggests that while MCAs have rather loose P2–P4 substrate specificity, the latter differs among different class members.

The ABPs designed from the substrate specificity screening are useful tools for detecting catalytically active proteoforms for bacterially produced CrMCA-II (Fig. 2, D and E). Theoretically, these ABPs may also be used to detect active CrMCA-II in vivo. However, we were thus far unable to find suitable microscopy conditions to differentiate the Cy5 signal between UVM4 and the *Crmca-ii* mutant, presumably due to low abundance of the mature protease in cells. An alternative explanation is the poor permeability of the Cy5 probe to the pool(s) of active CrMCA-II. Therefore, further optimization of the chemical structure of these ABPs to improve permeability, as well as screening for physiologically relevant conditions facilitating CrMCA-II activation, are both required for live imaging of CrMCA-II activity in cells.

### PM localization and the thermoprotective role of CrMCA-II

MCAs feature diverse subcellular localization patterns, but most are nucleocytoplasmic and can be found in both soluble (cytosolic) and insoluble (protein aggregates and endoplasmic reticulum [ER]) protein fractions (Huh 2022). Localization of CrMCA-II at the PM is thus uncommon and therefore intriguing. The only other known MCA with similar localization validated experimentally is TbMCA-Id (also named MCA4) from *T. brucei*, a type-I pseudopeptidase lacking catalytic Cys (Proto et al. 2011). Both CrMCA-II and TbMCA-Id lack transmembrane domain(s), pointing to their strong affinity for lipids and/or other membrane proteins. Accordingly, TbMCA-Id was previously shown to undergo *N*-myristoylation and palmitoylation in vivo to become associated with the internal surface of the flagellar membrane (Proto et al. 2011). Being devoid of a *N*-myristoylation motif, the CrMCA-II sequence yet contains a number of predicted palmitoylation motifs (Ning et al. 2020), which might contribute to the PM localization of the protein.

Interestingly, the presence of transmembrane domains is a widespread feature among orthocaspases and type-I MCAs in prokaryotes (Klemenčič and Funk 2018; La et al. 2022). Furthermore, transmembrane domains are predicted to occur (DeepTMHMM, Hallgren et al., 2022), albeit with much lower frequency, in type-I MCAs from unicellular green algae, including *A. protothecoides* 0710 and *Picochlorum* sp. It thus seems likely that direct association with the PM is an ancient feature of C14 proteases, which was preserved by prokaryotes and some single-celled eukaryotes but lost during the evolution of multicellularity. The latter assumption, however, is not completely true, since animal caspase-8 was shown to dimerize and become activated upon assembly of the death inducing signaling complex (DISC) at the PM during initiation of the extrinsic apoptosis pathway (Feig et al. 2007). Apart from its proteolytic role in apoptosis, caspase-8 promotes adhesion and Erk signaling in neuroblastoma cells via recruiting PM-associated Src tyrosine kinase (Finlay and Vuori 2007). Notably, this function of caspase-8 is independent of its catalytic activity, resembling the non-catalytic role of CrMCA-II revealed in the current study. Nonetheless, in contrast to CrMCA-II, both procaspase-8 (zymogen) and mature caspase-8 enzyme are present only in the cytosolic S-100 fraction and are absent in the light and heavy membrane fractions (Breckenridge et al. 2002; Fig. 5, B and D).

As only the full-length zymogen but not processed CrMCA-II is present at the PM, while the cytosol and the endomembranes contain both types of proteoforms (Fig. 5, B and D), there must be a tight spatial control over protease activity of CrMCA-II in the cell. Thus, the PM may serve as a compartment sequestering zymogen, whereas CrMCA-II protease maturation and substrate cleavage might take place in the cytoplasm. Given the critical requirement of Ca^2+^ for CrMCA-II activation and that the ER is a Ca^2+^ storage site with a high concentration (Stael et al. 2011), it is reasonable to assume that the proteolytic function of CrMCA-II (if any) is fulfilled in or around the ER when Ca^2+^ is released. However, testing a panel of knockout and complementation strains under a wide range of nutrient and environmental settings (Supplemental Fig. S13) failed to identify conditions wherein Chlamydomonas growth was dependent on the protease activity of CrMCA-II.

**Figure 6.**
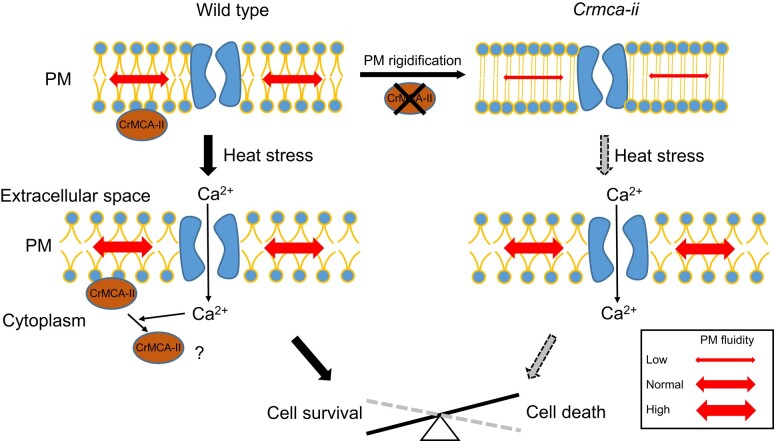
Hypothetical model for CrMCA-II-dependent regulation of thermotolerance. Under favorable growth conditions, CrMCA-II is preferentially present as a zymogen attached to the PM and prevents PM rigidification through an as yet unknown mechanism. HS elevates PM fluidity, the effect of which becomes more dramatic in the *Crmca-ii* cells with rigidified PM and compromising viability, accounting for their decreased thermotolerance. An increase in PM fluidity under HS stimulates influx of Ca^2+^ (Schroda et al. 2015) which, in turn, mediates the PM-to-cytoplasm translocation of CrMCA-II. The role of the cytoplasmic pool of CrMCA-II under HS and, in particular, whether it contributes to cytoprotection, remains unknown.

The PM plays a fundamental role in intra- and intercellular communication and in response to adverse environmental conditions, serving also as a crucial component of the cell repair machinery (Horn and Jaiswal 2019; Jaillais and Ott 2019). The non-proteolytic role of the PM-localized CrMCA-II zymogen in HS may be related to its ability to modulate PM fluidity, alleviating hypofluidity under normal conditions and, correspondingly, preventing a large-amplitude hyperfluidity response to high temperatures (see model in Fig. 6). Indeed, changes in PM fluidity have been shown to play a causative role in or correlate with various types of cell death in animals (summarized in Tekpli et al. 2013). In Chlamydomonas, treatment with the membrane rigidifier DMSO decreases cell viability (Haire et al. 2018). Accordingly, the hypofluid PM in *Crmca-ii* strains may fail to support transient lipid mobility after heat injury, undermining cell repair and leading to increased cell death (Horn and Jaiswal 2019).

In contrast to animals (Shuba 2021), the heat response in plants remains poorly characterized. It is postulated that both streptophytes and chlorophytes sense heat via an increase in PM fluidity causing activation of the cyclic nucleotide-gated ion channels and controlled entry of extracellular Ca^2+^ to the cytosol, which further leads to the heat shock factor (HSF)-mediated transcriptional response and biosynthesis of necessary proteins, such as heat shock proteins (HSPs) (Mittler et al. 2012; Cano-Ramirez et al. 2021; Guihur et al. 2022). We initially thought that the compromised thermotolerance of *Crmca-ii* strains was associated with aberrant HS sensing at the level of HSF and HSP production, but did not observe significant differences in the transcript levels of *HSF*s and major *HSP*s known to execute HS response in Chlamydomonas (Zhang et al. 2022a, b) between UVM4 and *Crmca-ii* (Supplemental Fig. S20). We therefore conclude that the mechanism of CrMCA-II-dependent thermoprotection is restricted to the regulation of physicochemical properties of the PM per se affecting its fluidity, bilayer stability, and hence functionality upon temperature shift (Horváth et al. 2008).

There are multiple factors underlying changes in PM fluidity, such as alterations in the conformation of integral membrane proteins, density and size of lipid rafts, and saturation level of phospholipids (Desai and Miller 2018; Pozza et al. 2022). Which of these factors are controlled or mediated by CrMCA-II will be addressed in our future work that will include characterization of the CrMCA-II interactome and, specifically, zymogen-containing megadalton-scale complex (Fig. 1G), as well as lipidomics analysis of the *Crmca-ii* strains. Intriguingly, CrMCA-II is predicted to harbor several intrinsically disordered segments, spanning the non-catalytic regions of the protein (predicted by flDPnn, Hu et al. 2021). Moreover, CrMCA-II was recently identified as a part of the heat-resistant intrinsically disordered proteome of Chlamydomonas (Zhang et al. 2018). This observation together with our findings that heat treatment stabilizes the zymogen of endogenous CrMCA-II and inhibits proteolytic activity of rCrMCA-II (Fig. 4, D to F) reinforce genetic evidence for a non-proteolytic role for rCrMCA-II in thermotolerance.

Interestingly, HS induces PM-to-cytoplasm translocation of CrMCA-II, in a Ca^2+^ influx-dependent manner, but independently of the catalytic residue Cys-141 (Figs. 5, C to F and 6, Supplemental Fig. S17, A and B). According to recent structural data obtained for AtMCA-IIa (Zhu et al. 2020), Ca^2+^ can bind to the high affinity-binding pocket of CrMCA-II (Asp-95, Asp-97, Glu-98, and Asp-100; Supplemental Fig. S3), and imposes structural changes causing its detachment from the PM. Whether PM-to-cytoplasm translocation of CrMCA-II has any functional role in HS response remains an open question. What role do cytosolic and EM-associated pools of proteolytically active CrMCA-II play under normal conditions is another conundrum calling for further optimization of CrMCA-II-specific ABPs and generation of genetically-encoded sensors of CrMCA-II protease activity (Fernández-Fernández et al. 2019) that would allow its noninvasive monitoring with subcellular resolution in vivo.

In conclusion, CrMCA-II, the single type-II MCA of Chlamydomonas, confers thermotolerance via a mechanism governing PM fluidity, which is completely independent of its protease activity. In this respect, the non-catalytic mode of CrMCA-II action is reminiscent of that of ScMCA-I, the single MCA encoded by the budding yeast genome, in suppressing protein aggregate formation (also under HS) and replicative aging, although the catalytic Cys-276 mutant of yeast MCA1 could only partially rescue *mca1* deletion phenotypes (Lee et al. 2010; Hill et al. 2014). Taken together, the findings in budding yeast and Chlamydomonas suggest an evolutionary scenario wherein the ancient primordial role of MCAs was to carry out quality control both in the cytoplasm (e.g. to counteract protein aggregation) and at the cell surface (e.g. to maintain membrane fluidity) for fitness and survival of unicellular organisms in fluctuating environments. Complete or partial independence of intricately regulated proteolytic activity may provide additional robustness to the proposed role of MCAs.

## Materials and methods

### Strains and culture conditions

Unless stated otherwise, The Chlamydomonas (*C. reinhardtii*) strain UV mutagenesis strain 4 (UVM4), with high efficiency to overexpress foreign genes (Neupert et al. 2009), was used as a control strain. All strains used in this study are listed in Supplemental Data Set 3. Chlamydomonas cells were grown in axenic TAP medium at 23 °C and under a light–dark cycle of 16 h light/8 h darkness with a light intensity of 110 *μ*mol m^−2^ s^−1^. The cells were harvested at a density of 1 to 2 × 10^7^ cells mL^−1^ unless stated otherwise.

### Phylogenetic analysis

The MCA sequences of chlorophytes and streptophytes were obtained from Phytozome 13 (https://phytozome.jgi.doe.gov/pz/portal.html) (Supplemental Files 1 and 2). The software MEGA X (Kumar et al. 2018) was used to perform multiple protein sequence alignment with the embedded MUSCLE program; the phylogenetic tree was reconstructed with default setting using the neighbor-joining method.

### rCrMCA-II production and purification

Recombinant 6*×*His-tagged CrMCA-II (rCrMCA-II) was produced using a codon-optimized construct in *Escherichia coli* and then purified through affinity chromatography and SEC as described (Sabljić et al. 2022). All plasmids used in this study are provided in Supplemental Data Set 4.

### Protease activity assay of rCrMCA-II

The proteolytic activity of 5 ng purified rCrMCA-II was measured at 25 °C in reaction buffer (optimal buffer conditions: 50 mM Tris-HCl, pH 7.5, 25 mM NaCl, 20 mM CaCl_2_, 0.1% [w/v] CHAPS, and 7.5 mM DTT), with 50 *μ*M Val-Arg-Pro-Arg-AMC (VRPR-AMC) as a substrate, unless stated otherwise. Fluorescence intensity was measured every min using a BMG Labtech POLARstar Omega Microplate Reader at an excitation or 360 to 380 nm and an emission of 440 to 460 nm. Cleavage activity was determined based on the slope of at least 10 recordings.

### Active site titration of CrMCA-II variants

The CrMCA-II variants were subjected to production in *E. coli* BL21 cells and subsequently purified with a Dynabeads His-Tag Isolation & Pulldown kit (ThermoFisher Scientific) according to the manufacturer's instructions. The eluates obtained with 300 mM imidazole were desalted using Vivaspin ultrafiltration spin columns (Cytiva). Titration of the CrMCA-II variants was performed using the peptidic substrate z-VRPR-fmk (Bachem) according to Hachmann et al. (2012).

### rCrMCA-II protease activity assay in native gel electrophoresis

To perform native electrophoresis, a 4% to 20% gradient stain-free gel was loaded with 7 *μ*g protein aliquots that had been incubated with 7.5 mM DTT for 15 min at 4 °C. Controls were treated the same way, but without DTT. The gel was then run at 150 V for 45 min in Tris/glycine buffer. After electrophoresis, 3 mL of DTT-free reaction buffer was added on top of the gel at room temperature and fluorescence was detected immediately using a BioRad Gel Doc EZ Imager programmed for ethidium bromide. All uncropped gel and membrane images are provided in Supplemental Fig. S21.

### SEC of total Chlamydomonas protein extracts

Cells were harvested in the late log phase from 100 mL TAP medium and suspended in 1 mL extraction buffer (50 mM Tris-HCl, pH 7.5, 25 to 100 mM NaCl, 1% [v/v] glycerol, 1 *μ*M aprotinin, 10 *μ*M pepstatin A, 1 mM PMSF, and 2 mM pefabloc SC). The cell mixture was sonicated for 3 min by 10 s pulses, with 5 s intervals (Vibra-Cell ultrasonic processor; Sonics & Materials, Inc.); the cell debris were pelleted by centrifugation at 17,000 × *g* for 20 min at 4 °C. The resulting crude cell extract was filtered through a 0.45 *μ*m filter before loading onto a HiLoad 16/60 Superdex 200 pg column (GE Healthcare) at 4 °C. A buffer containing 50 mM Tris-HCl (pH 7.5) and 25 mM NaCl was used for equilibration and elution, and elution profiles were recorded at 280 nm. Fractions of 750 *μ*L each were collected, precipitated with 90% (v/v) methanol (Bychkov et al. 2011), and dissolved in 40 *μ*L 1 × SDS sample buffer. Immunoblotting was performed after separating 10 *μ*L of each fraction by SDS-PAGE on a 4% to 20% gel. A set of protein standards was used as a reference for calibration and determination of the molecular mass of CrMCA-II protein species in the samples.

### Chemical reagents for organic synthesis

All chemicals used for the synthesis of substrates and ABPs were purchased from commercial suppliers and used without purification unless otherwise noted. Rink amide RA resin (loading 0.48 mmol g^−1^) was used for the synthesis of ACC-labeled substrates, and 2-chlorotrityl chloride resin (1.59 mmol g^−1^, 100 to 200 mesh) was used for the synthesis of peptides that were further converted into ABPs (both resins were from Iris Biotech GmbH, Germany). Fmoc-protected amino acids (all > 98% pure) were purchased from various suppliers: Iris Biotech GmbH, Creosalus, P3 BioSystems, QM Bio, Bachem. N,N-diisopropylethylamine (DIPEA, peptide grade), diisopropylcarbodiimide (DICI, peptide grade), piperidine (PIP, peptide grade), and trifluoroacetic acid (TFA, purity 99%) were all from Iris Biotech, GmbH. 2,4,6-Trimethylpyridine (2,4,6-collidine, peptide grade), triisopropylsilane (TIPS, purity 99%), 2,2,2-trifluoroethanol (TFE), anhydrous tetrahydrofuran (THF), hydrogen bromide (30% [w/v] in acetic acid), 4-methylmorpholine (NMM), isobutylchloroformate (IBCF), and 2,6-dimethylbenzoic acid (2,6-DMBA) were all purchased from Sigma Aldrich. N-hydroxybenzotriazole (HOBt, monohydrate) was from Creosalus. HATU and HBTU (both peptide grade) were from ChemPep Inc. N,N′-dimethylformamide (DMF, peptide grade) and acetonitrile (ACN, HPLC pure) were from WITKO Sp. z o.o., Poland. Methanol (MeOH, pure for analysis), dichloromethane (DCM, pure for analysis), diethyl ether (Et_2_O, pure for analysis), acetic acid (AcOH, 98% pure), and phosphorus pentoxide (P_2_O_5_, 98% pure) were from POCh, Poland. Cyanine-5 NHS was purchased from Lumiprobe. Diazomethane was generated according to the Aldrich Technical Bulletin (AL-180) protocol.

### Characterization of rCrMCA-II P4–P2 substrate specificity using HyCoSuL

To determine the specificity of CrMCA-II for the P4–P2 positions, the P1-Arg HyCoSuL library was used (Poreba et al. 2017). P4, P3, and P2 sub-libraries were each screened at 100 *μ*M final concentration in 100 *μ*L final volume with rCrMCA-II. The active enzyme was in the 2 to 10 nM range, depending on the sub-library used. The screening time was 30 min, but for each substrate cleavage, only the linear portion of the progression curve (10 to 15 min) was used for analysis (RFU s^−1^ calculation) to avoid problems of substrate consumption. Screening of each library was performed at least in triplicate, and the average value was used to create the rCrMCA-II specificity matrix (the standard deviation [Sd] for each substrate was below 15%). The hydrolysis rate for the best recognized amino acid at each position was set to 100%, and other amino acids were adjusted accordingly.

### Synthesis and screening of individual optimized substrates

ACC-labeled tetrapeptide substrates were synthesized and purified as described elsewhere (Poręba et al. 2014). Substrate hydrolysis at a concentration of 100 *μ*M was carried out for 30 min. rCrMCA-II concentration was 2.1 nM. Each experiment was performed in triplicate and the rate of substrate hydrolysis (RFU s^−1^) was presented as an average value (Sd for each substrate hydrolysis was below 15%).

### Enzyme kinetics

All kinetics experiments were performed using a Gemini XPS (Molecular Devices) plate reader operating in the fluorescence kinetic mode using 96-well plates (Corning, Costar). ACC-labeled fluorescent substrates were screened at 355 nm (excitation) and 460 nm (emission) wavelengths under optimal buffer conditions. Buffer was prepared at an ambient temperature and all kinetics measurements were performed at 37 °C. The kinetic parameters (*k*_cat_, *K_M_*, *k*_cat_/*K_M_*) for ACC-labeled substrates were determined using Michaelis–Menten kinetics according to the protocol described by Poręba et al. (2014). All experiments were performed at least in triplicate and the results were presented as average values (Sd for all tested substrates was below 15%).

### Synthesis of fluorescent ABPs

Cy5-labeled irreversible ABPs probes for rCrMCA-II were synthesized and purified as described previously (Poreba et al. 2018). In brief, Boc-Ahx-peptide-COOH was synthesized on solid support using 2-chlorotrityl chloride resin and used without further purification. In parallel, the Boc-Arg(Boc)_2_-AOMK warhead was synthesized through generation of diazomethane and reaction with mixed anhydrides (Boc-Arg(Boc)_2_-OH → Boc-Arg(Boc)_2_-CH_2_N_2_), followed by the conversion of the crude product into bromomethyl ketone (Boc-Arg(Boc)_2_-BMK) and, finally, into acyloxymethyl ketone (Boc-Arg(Boc)_2_-AOMK). Next, the crude product was de-protected in TFA/DCM mixture, and the NH_2_-Arg-AOMK was coupled with Boc-(linker)-peptide-COOH to yield Boc-(linker)-peptide-Arg-AOMK. The product was purified by HPLC, and after Boc de-protection, it was labeled with Cy5 to obtain Cy5-Ahx-peptide-Arg-AOMK. The final products were purified by HPLC, analyzed via HR-MS and HPLC and dissolved in DMSO (10 mM).

### ABP binding assay

Each reaction contained 0.4 *μ*M rCrMCA-II or rCrMCA-II^C141A^ and the indicated concentration of ABP, which was co-incubated for 5 min. The reaction was stopped by adding SDS sample buffer. Two-hundred nanograms of purified protein was separated by SDS-PAGE, and the gel was then scanned at 700 nm using an Odyssey infrared scanner (LI-COR Biosciences). Total protein was visualized on a ChemiDoc XRS+ imaging System after staining with Oriole fluorescent gel stain solution (Bio-Rad) for 1.5 to 2 h.

### Genome editing

A CRISPR/Cas9 based targeted insertional mutagenesis approach (Picariello et al. 2020) was used to knock out the *CrMCA-II* gene. A single guide RNA (sgRNA) was designed for targeting the exon region flanking the sequences encoding the catalytic dyad to disrupt *CrMCA-II*. Electroporation was used to introduce a donor DNA comprising 40 bp of flanking sequences on either side of an expression cassette consisting of the *Hsp70/RBCS2* synthetic *promoter*, the *AphVIII* selection gene, the *RBCS2* terminator, and the ribonucleoprotein complex. The resulting transformants were screened by colony PCR and confirmed by immunoblotting with an anti-CrMCA-II antibody.

### Genomic DNA isolation

Ten mL of cells grown in TAP medium was harvested and resuspended in 150 *μ*L H_2_O and 300 *μ*L of SDS-EB buffer (2% [w/v] SDS, 400 mM NaCl, 40 mM EDTA, 100 mM Tris-HCl, pH 8.0). The cell suspension was mixed with 350 *μ*L phenol:chloroform:isoamyl alcohol (25:24:1, v/v/v) by inverting for a few min, which led to the separation of 2 phases after centrifugation at 2,000 × *g* for 5 min at room temperature. The upper aqueous phase was extracted using 300 *μ*L chloroform:isoamyl alcohol (24:1, v/v). The resulting aqueous phase was mixed with 2 volumes of absolute ethanol and stored at −80 °C for 30 min. The genomic DNA was pelleted by centrifugation at 17,000 × *g* for 10 min at room temperature and washed twice with 200 *μ*L 70% (v/v) ethanol. The genomic DNA was then air-dried and dissolved in H_2_O.

### RNA isolation, qPCR, and reverse transcription quantitative PCR (RT-qPCR)

Total RNA was isolated using a Spectrum Plant Total RNA Kit (Sigma-Aldrich) according to the manufacturer's instructions. DNAse I was added during isolation to remove any remaining genomic DNA. First-strand complementary DNA (cDNA) was generated using Maxima Reverse Transcriptase (ThermoFisher). For qPCR analysis, 200 ng of genomic DNA or cDNA reverse transcribed from 12.5 ng of total RNA was used in each reaction. The relative abundance of target genes was calculated using the 2^−ΔΔCt^ method (Livak and Schmittgen 2001) with *RACK1* (Mus et al. 2007) as a reference for normalization.

### Proteolytic activity assay of Chlamydomonas cell lysates and SEC fractions of total protein extracts

To prepare cell lysates, 10 mL of cells at the density of ∼1 × 10^7^ cells mL^−1^ was harvested and stored at −80 °C. Before extraction, the cells were thawed on ice for 5 min. Each sample was then suspended in 150 *μ*L of extraction buffer (50 mM Tris-HCl, pH 7.5, 25 to 100 mM NaCl, 1 mM EDTA, 1% [v/v] glycerol, 1 *μ*M aprotinin, 10 *μ*M pepstatin A, 1 mM PMSF, and 2 mM pefabloc SC) and sonicated for 30 s by 10 s pulses, with 5 s intervals, and the cell debris was pelleted at 7,800 × *g* for 10 min at 4 °C. The resulting supernatant (cell lysate) was used for the assay. The proteolytic activity of cell lysates was measured in 50 *μ*L extraction buffer supplemented with 0.1% (w/v) CHAPS, 7.5 mM DTT, 20 mM CaCl_2_, 50 *μ*M of either VRPR-7-amino-4-methylcoumarin (AMC) or HRTR-ACC, and 1 *μ*L cell lysate at 25 °C. SEC fractions were prepared as described above, pooled by 3 fractions, and the proteolytic activity was measured in 50 *μ*L elution buffer (50 mM Tris-HCl [pH 7.5] and 25 mM NaCl) supplemented with 0.1% (w/v) CHAPS, 7.5 mM DTT, 50 *μ*M of VRPR-AMC, and 10 *μ*L SEC fractions, with or without addition of 20 mM CaCl_2_ at 25 °C. The fluorescent signal was detected on a POLARstar Omega Plate Reader (BMG LABTECH).

### Y2H assay

The Y2H experiments were performed as previously described (Zou et al. 2020). The full-length coding sequences of *CrMCA-II* and *Serpin* were cloned into the GAL4 AD or/and BD vectors. The BD-CrMCA-II vector was used as a template to construct BD-CrMCA-II^C141A^. The Y2H assays were performed according to the Matchmaker Gold Yeast Two-Hybrid System (Takara). The Y2HGold strain was co-transformed with the AD and BD vectors; the transformants were selected on synthetic defined (SD) medium −Leu/−Trp. The transformed colonies were spotted onto SD medium −Ade/−His/−Leu/−Trp plates and were grown at 30 °C for 5 d before photographs were taken.

### Chlamydomonas subcellular fractionation

We followed previously published procedures (Norling et al. 1996; Herbik et al. 2002) with some modifications (see Supplemental Fig. S16). All steps were performed at 4 °C. Briefly, cells were harvested at 4,000 × *g* for 5 min and snap-frozen in liquid nitrogen. One to 2 g cells were mixed with 40 mL extraction buffer (250 mM sucrose, 50 mM HEPES pH 7.5, 1 mM EGTA, 1 mM KCl, 0.6% [w/v] PVPP, 0.1 mM PMSF, and 2.5 mM DTT) for 5 min on ice before sonicating twice for 10 min on ice (500 W, 20% amplitude; Vibra-Cell ultrasonic processor; Sonics & Materials, Inc.). The unbroken cells, chloroplasts and cell debris were removed by centrifugation for 3 min at 1,200 × *g*. The first supernatant (S1) was centrifuged for 5 min at 13,000 × *g* to remove the pellet containing mainly mitochondria and thylakoids, and to collect the second supernatant (S2) containing LDMs from the Golgi, the ER, the tonoplast, as well as the PM. The S2 supernatant was centrifuged at 4 °C for 2 h at 68,000 × *g* to obtain the LDM-containing pellet (P3) and the S3 supernatant. P3 was resuspended in a maximum of 2.5 mL resuspension buffer (250 mM sucrose, 5 mM potassium phosphate pH 7.8, and 5 mM DTT), and the PM vesicles were separated from the endomembranes by a 2-phase partitioning system. The P3 suspension (2.25 g) was loaded onto an ice-cold polymer mixture (6.75 g) comprised of 6.4% (w/v) dextran T500, 6.4% (w/v) polyethylene glycol (PEG, molecular weight of 4,000), 250 mM sucrose, 5 mM potassium phosphate (pH 7.8), and 5 mM KCl. After centrifugation at 2,000 × *g* for 10 min, the mixture was separated into 2 phases. The upper PEG phase, containing the PM fraction, was further partitioned against a fresh lower phase to increase the purity of the PM fraction. Subsequently, the PEG and dextran phases were diluted 5-fold with dilution buffer (0.33 M sucrose, 5 mM MOPS pH 7.0, 1 mM EDTA, and 2 mM DTT). The PM proteins and the endomembrane proteins were pelleted from the PEG phase and the dextran phase, respectively by centrifugation at 68,000 × *g* for 1 h at 4 °C. The endomembrane and the PM proteins were resuspended in dilution buffer for immunoblotting.

### Protein extraction and immunoblotting

Two mL of cell cultures in late log phase (1 to 2 × 10^7^ cells mL^−1^) were harvested by centrifugation at 17,000 × *g* for 1 min at room temperature and were snap-frozen in liquid nitrogen. The cell pellet was dissolved in 200 *μ*L 1 × SDS sample buffer (50 mM Tris-HCl pH 7.5, 100 mM DTT, 2% [w/v] SDS, 0.1% [w/v] bromophenol blue, 10% [w/v] glycerol) and incubated at 70 °C for 5 min. The samples were vortexed at the highest speed and then centrifuged at 17,000 × *g* for 10 min at room temperature. The resulting supernatant was used for immunoblotting. The anti-CrMCA-II antibody was generated by Agrisera using a peptide antigen and characterized in Supplemental Fig. S12A. The anti-HA (Y-11) antibody was from Santa Cruz Biotechnology (sc-805). The anti-H^+^ ATPase antibody was from Agrisera (AS07 260). The membranes were blocked in 5% (w/v) nonfat-dried milk dissolved in Tris-buffered saline with 0.1% (v/v) Tween 20 (TBST). Anti-CrMCA-II, anti-HA, anti-H^+^ ATPase antibodies, and a secondary anti-rabbit antibody were diluted 1:1,000, 1:2,000, 1:5,000, and 1:10,000, respectively. Protein molecular weight markers were Precision Plus Protein Dual Color Standards (Biorad, 1610374). The PVDF membranes were stained with Ponceau S and destained with distilled water.

### Phenotyping screen

The Chlamydomonas strains used for phenotypic screening were first cultured until similar cell density was achieved in the log phase. The same amounts of cells with serial dilutions (1, 1:10, and 1:100) were spotted onto plates with different nutrient composition. The spots were dried under a sterile hood before the plates were sealed with parafilm. Afterwards, the plates were kept in an incubator at 23 °C with a 16 h light/8 h dark cycle and light intensity of 110 *μ*mol m^−2^ s^−1^. For heat treatment, 100 *μ*L cells at log phase (5 to 6 × 10^6^ cells mL^−1^) were exposed to 42 °C for 2 h. To recover from HS, 5 *μ*L of the culture was inoculated on plates. Alternatively, 50 *μ*L of the culture was transferred to 3 mL fresh TAP medium.

### Cell death measurements

Cells in log phase (∼5 × 10^6^ cells mL^−1^) were subjected to HS at 42 °C for 1 h and then cooled down to room temperature before being treated with fluorescein diacetate (FDA, final concentration 2 *μ*g mL^−1^; Minina et al. 2013). Cell death was checked immediately after HS. The FDA-treated cells were loaded into a hemocytometer and photographed. When excited at 480 nm, living cells appear green, while dead cells appear red because of chlorophyll autofluorescence. Cell death was calculated using the following formula: cell death (%) = dead cells/(dead cells + living cells) ∗ 100%.

### Live imaging of *CrMCA-II*

The *CrMCA-II* gene was cloned into the MoClo toolkit (Crozet et al. 2018). Subsequently, a plasmid containing *PsaD* promoter, the genomic sequence of CrMCA-II, the mVenus gene, and the PsaD terminator was assembled to overexpress CrMCA-II-mVenus. The transformants at log phase were screened and imaged by confocal microscopy (Zeiss LSM 780), with the excitation and emission at 514 nm and 540 to 580 nm, respectively.

### Fluorescence recovery after photobleaching (FRAP)

Due to the high sensitivity to photobleaching upon 42 °C HS resulting in cell damage, the temperature of HS for the FRAP analysis was lowered to 39 °C. The cells were labeled with DiOC6(3) (Invitrogen, final concentration 0.05 *μ*g mL^−1^) for 1 min at room temperature, followed by 2 washes with fresh TAP medium. The labeled PM was observed by confocal microscopy (LSM 780, Carl Zeiss), with the excitation at 488 nm and the emission at 501 to 570 nm. FRAP analysis was conducted as described (Gutierrez-Beltran et al. 2021), with the following modifications: 60 iterations, 1 s per frame, and 75% transmittance with the 488 nm laser lines of the argon laser. Pre- and post-bleach scans were performed using the laser power at 0.5% to 2% transmittance for 488 nm and 0% for all other laser lines.

### Statistical analysis

Statistical analyses were performed as described in each figure legend. Statistical data are provided in Supplemental Data Set 5

### Accession numbers

The genes studied in this work can be found in Phytozome (https://phytozome.jgi.doe.gov/pz/portal.html) with the following accession numbers: *CrMCA-II* (Cre03.g184700); *CrMCA-I* (Cre12.g517451); *Serpin* (Cre16.g679550); *HSF1* (Cre09.g387150); *HSP22A* (Cre07.g318800); and *HSP90A* (Cre09.g386750).

## Supplementary Material

koad289_Supplementary_Data

## Data Availability

The data underlying this article are available in the article and in its online supplementary material.

## Supplemental data

The following materials are available in the online version of this article.

**Supplemental Figure S1**. Phylogeny of MCAs from the chlorophyte and streptophyte lineages.

**Supplemental Figure S2.** Biochemical characteristics of rCrMCA-II.

**Supplemental Figure S3.** Predicted structure of CrMCA-II.

**Supplemental Figure S4.** Effect of DTT and H_2_O_2_ on the activity of CrMCA-II.

**Supplemental Figure S5.** Yeast two-hybrid assay of the interaction between full-length CrMCA-II or its catalytically inactive mutant CrMCA-II^C141A^ and different protein regions.

**Supplemental Figure S6.** Sequence alignment of type-II MCAs from Chlamydomonas and Arabidopsis.

**Supplemental Figure S7.** Immunoblot and proteolytic activity analyses of SEC fractions from Chlamydomonas protein extracts.

**Supplemental Figure S8.** Substrate specificity of rCrMCA-II in the P4–P2 positions.

**Supplemental Figure S9.** Structures of ACC-labeled tetrapeptide substrates for CrMCA-II.

**Supplemental Figure S10.** Structures and MS data of Cy5-labeled ABPs for CrMCA-II.

**Supplemental Figure S11.** Sequence alignment of a part of exon 4 of *CrMCA-II* genomic DNA in control (UVM4) and mutant (*Crmca-ii-9* and *Crmca-ii-23*) strains.

**Supplemental Figure S12.** Characterization of *CrMCA-II* mutants using a specific anti-CrMCA-II antibody and substrate cleavage assays.

**Supplemental Figure S13.** Phenotyping of UVM4, *Crmca-ii* mutant (*Crmca-ii-9* and *Crmca-ii-23*), and complementation (*Crmca-ii-23 CrMCA-II-3×HA* and *Crmca-ii-23 CrMCA-II^C141A^-3×HA*) strains grown on plates under various nutrient and environmental conditions.

**Supplemental Figure S14.** Algal strains 6 d after recovery from a 2 h HS treatment at 42 °C.

**Supplemental Figure S15.** Subcellular localization of mVenus and CrMCA-II-mVenus in strain CC-4533 under normal conditions.

**Supplemental Figure S16.** Procedure for subcellular fractionation used for CrMCA-II and H^+^ATPase immunoblot analysis shown in Fig. 5, B and D.

**Supplemental Figure S17.** Confocal microscopy analysis of CrMCA-II localization.

**Supplemental Figure S18.** FRAP analysis.

**Supplemental Figure S19.** Selected time frames from FRAP analysis shown in Fig. 5G.

**Supplemental Figure S20.** RT-qPCR analysis of HS-responsive gene expression.

**Supplemental Figure S21.** Uncropped gel and membrane images.

**Supplemental Table S1.** List of activity-based probes generated and used in this study.

**Supplemental Data Set 1.** Mass spectrometry analysis of ACC-labeled tetrapeptide substrates of CrMCA-II.

**Supplemental Data Set 2.** List of primers used in this study.

**Supplemental Data Set 3.** List of strains used in this study.

**Supplemental Data Set 4.** List of plasmids used in this study.

**Supplemental Data Set 5.** Summary of statistical analyses from this study.

**Supplemental File 1.** Sequence alignment.

**Supplemental File 2.** Phylogenetic tree data.
