# Supplementary material for: Thermoprotection by a cell membrane–localized metacaspase in a green alga
Source: Plant Cell. 2023 Nov 16;36(3):665–87. doi: 10.1093/plcell/koad289 (PMC10896300; doi:10.1093/plcell/koad289)
Supplement: koad289_Supplementary_Data [file koad289_supplementary_data.zip › TPC2023RA00478R1_Supplemental_Figures_Tables.pdf]

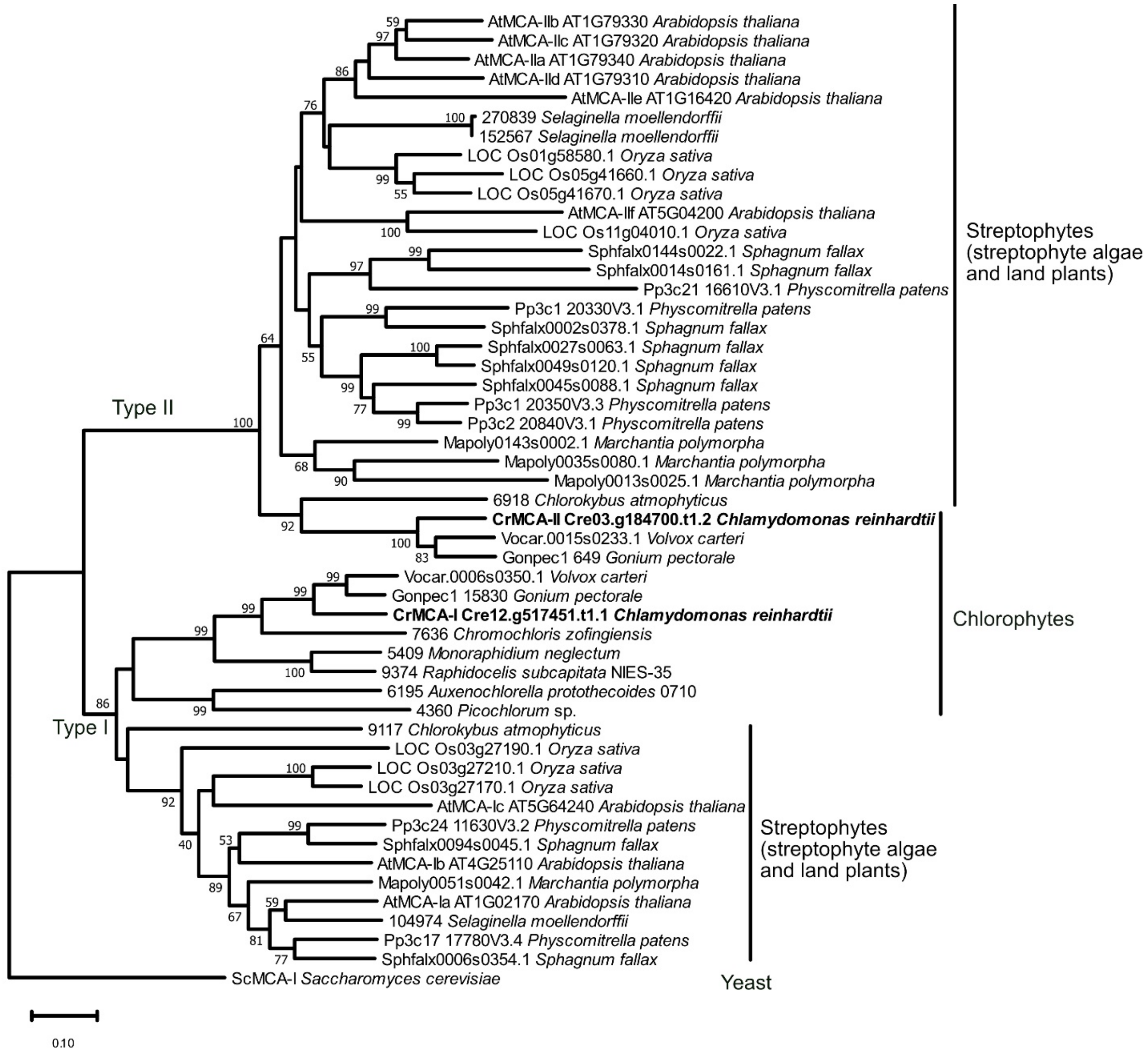

Supplemental Figure S1. Phylogeny of MCAs from the chlorophyte and streptophyte lineages.
Supports Figures 1 and 3.

Each sequence contains the accession number from Phytozome 13 (https://phytozomenext.jgi.doe.gov/). The sequences from *Chlamydomonas reinhardtii* are highlighted in bold. A type-I MCA from budding yeast (*Saccharomyces cerevisiae*) is included and set as outgroup. MEGA X was used for alignment and phylogenetic analysis, with default settings of the neighbor-joining method. A thousand bootstrap replicates were run; percentage support for each branch over 50 are displayed. The scale bar represents the amino acid substitution rate per site. The sequence alignment is shown in Supplemental File S1. The machine-readable tree data is shown in Supplemental File S2.

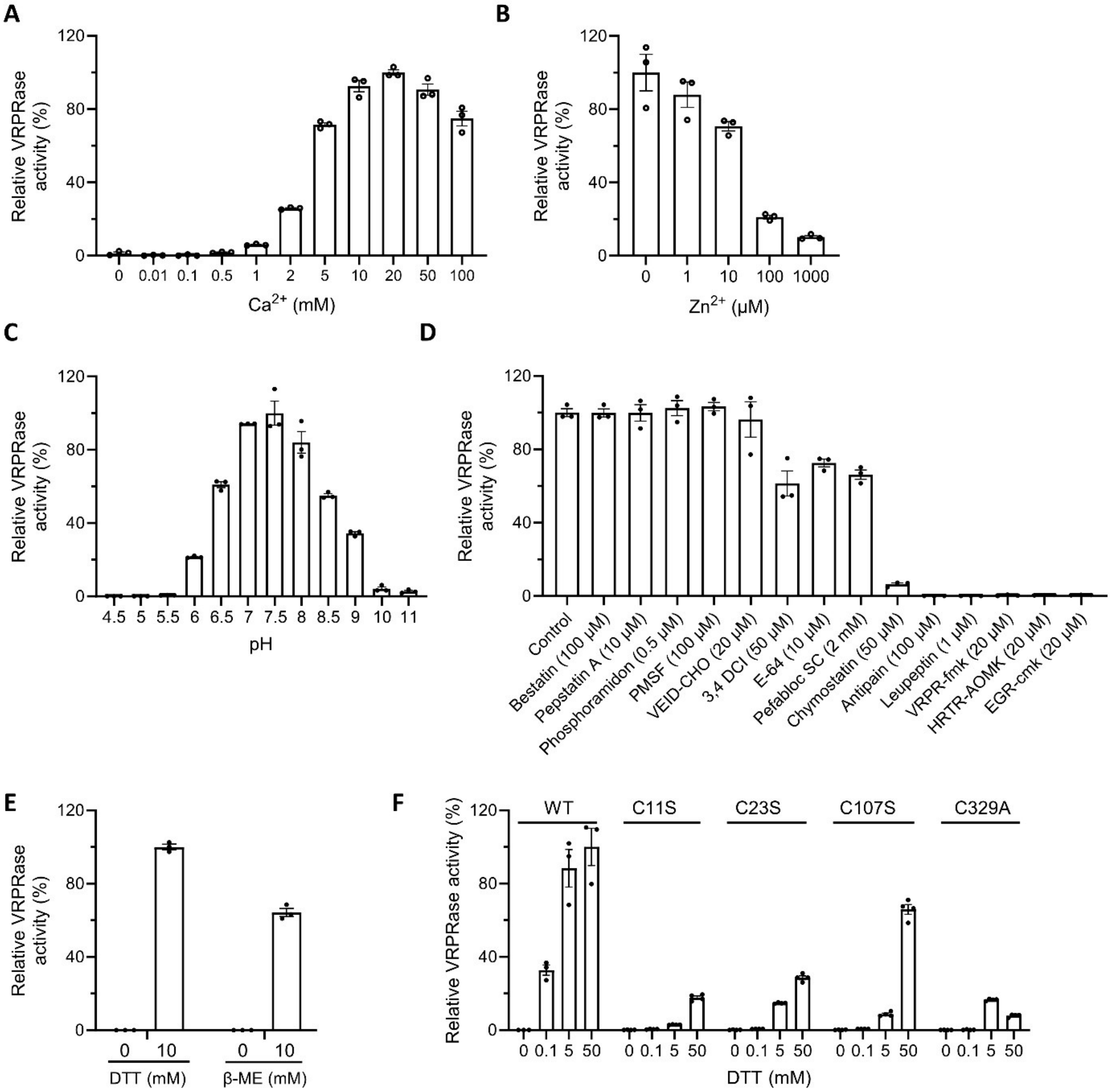

Supplemental Figure S2. Biochemical characteristics of rCrMCA-II.

Supports Figure 1.

Effects of Ca^{2+} ($CaCl_2$) (**A**), zinc ($ZnCl_2$) (**B**), pH (**C**), protease inhibitors (**D**), and reducing agents (**E**) on the proteolytic activity of rCrMCA-II under optimal buffer conditions (50 mM Tris-HCl, pH 7.5, 25 mM NaCl, 20 mM $CaCl_2$, 0.1% [v/v] CHAPS and 7.5 mM DTT, unless stated otherwise) using Ac-VRPR-AMC as a substrate.

(**F**) Activity of rCrMCA-II variants, compared to wild-type (WT) rCrMCA-II (data from Figure 1B), with increased concentration of DTT.

Data represent the means ± SEM of triplicate measurements.

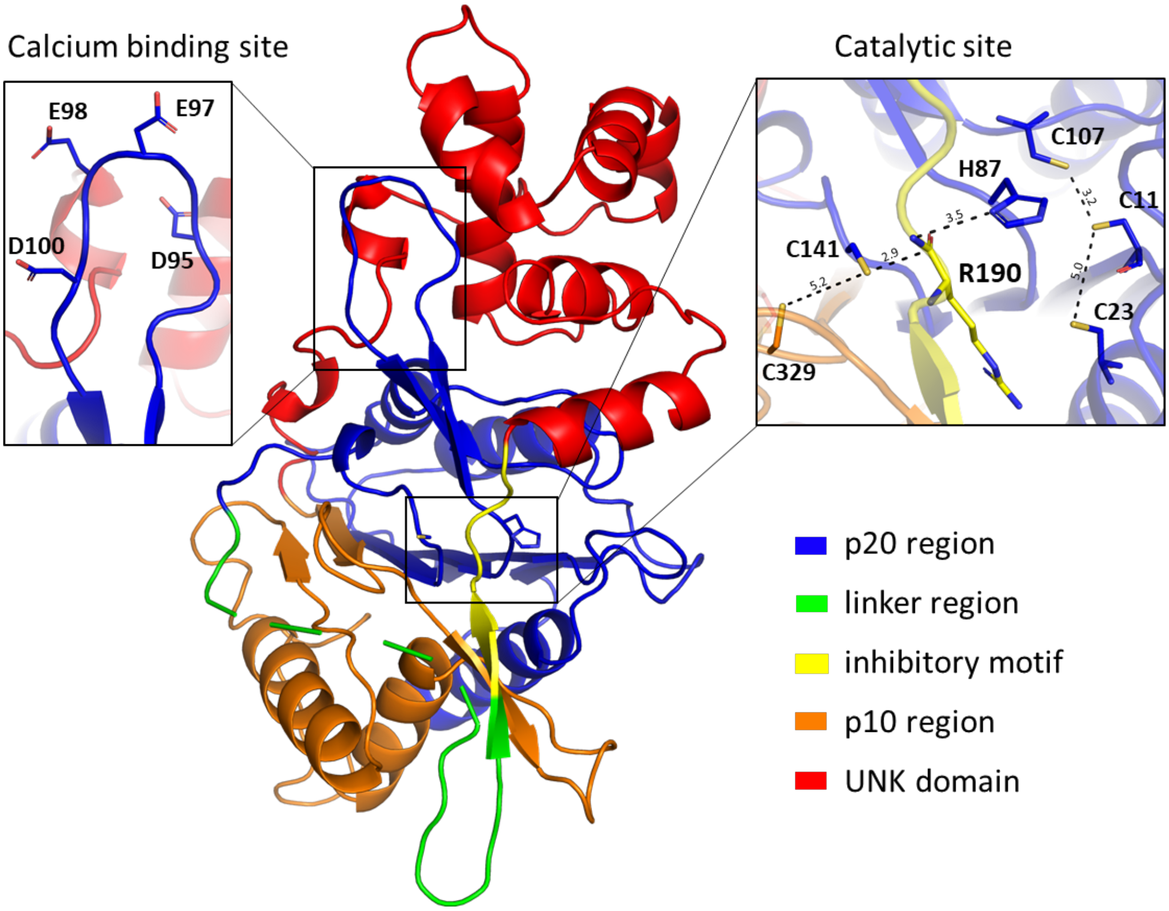

Supplemental Figure S3. Predicted structure of CrMCA-II.
Supports Figure 1.
The model was obtained using the Phyre2 web portal (Kelley et al., 2015) in One-to-One threading mode with the AtMCA-IIa structure. UNK (unknown) domain was previously included into the linker region of type-II MCAs, but is now defined as a separate structural element (Stael et al., 2023). The conformations of the Cys side chains in the model were slightly adjusted to emphasize the potential for forming Cys–Cys bridges.

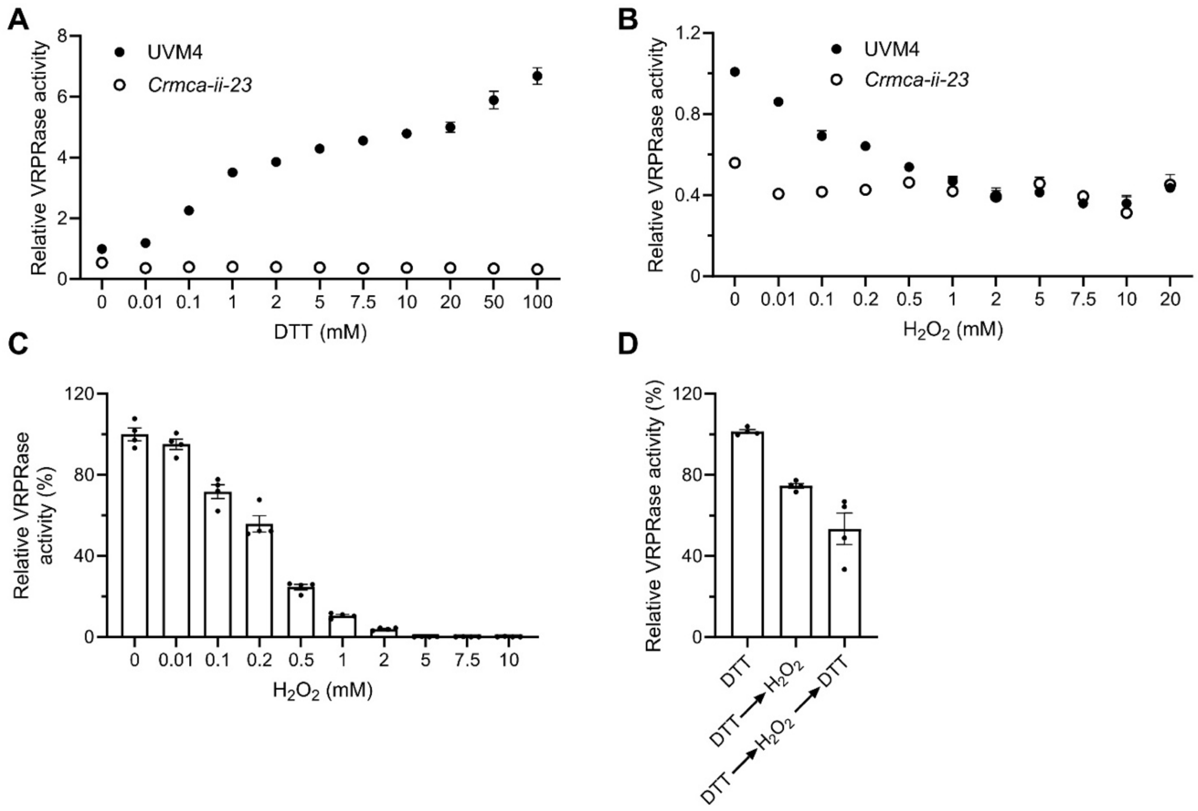

Supplemental Figure S4. Effect of DTT and H_2O_2 on the activity of CrMCA-II.
Supports Figure 1.
(**A, B**) Effect of increased concentration of DTT (**A**) or H_2O_2 (**B**) on VRPRase activity in cell lysates prepared from control (UVM4) and CrMCA-II-deficient (*Crmca-ii-23*) strains of Chlamydomonas. Proteolytic activity was measured under optimal buffer conditions (except varying concentration of DTT in **A**) using Ac-VRPR-AMC as a substrate.
(C) Effect of H_2O_2 on VRPRase activity of rCrMCA-II in optimal buffer with 7.5 mM DTT and 20 mM $CaCl_2$.
(D) Effect of re-addition of DTT on VRPRase activity from rCrMCA-II following its inhibition by H_2O_2. The activity was first monitored in the presence of 7.5 mM DTT (indicated as DTT). After a 2-min reaction, 0.2 mM H_2O_2 (1 µL of a 10-mM stock) was added, and the activity was monitored again (indicated as DTT→H_2O_2). After another 2-min reaction, 50 mM DTT (1 µL of a 2.5-M stock) was added and the substrate cleavage was monitored for another 2-min interval (indicated as DTT→H_2O_2→DTT). The activity with 7.5 mM DTT was set to 100%.
Data represent the means ± SEM of triplicate (**A** and **B**) or quadruplicate (**C** and **D**) measurements.

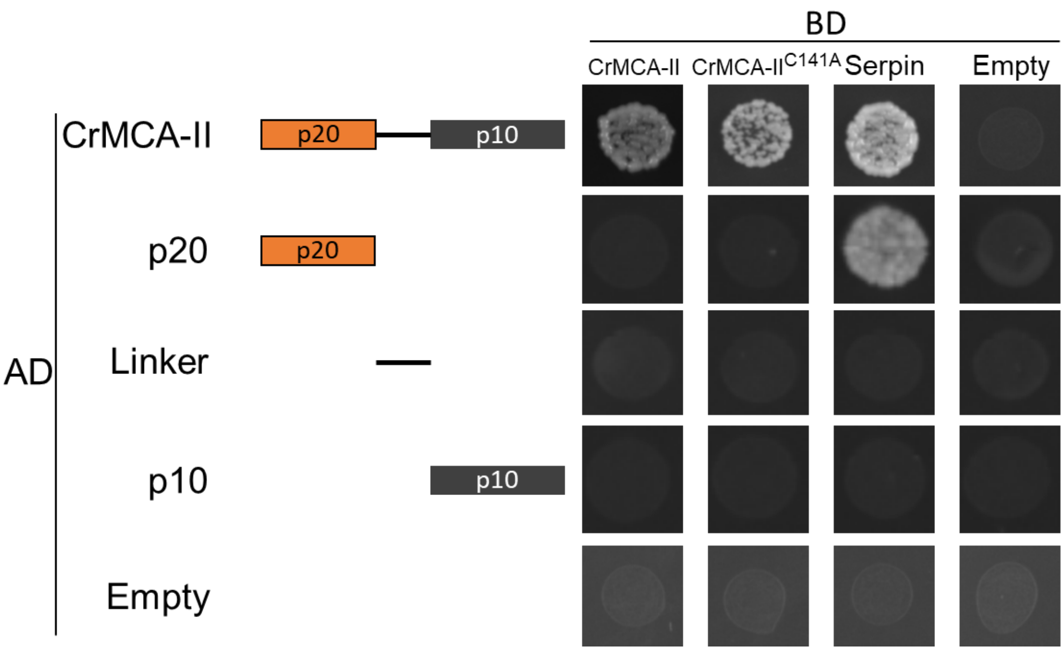

Supplemental Figure S5. Yeast two-hybrid assay of the interaction between full-length CrMCA-II or its catalytically inactive mutant CrMCA-II^{C141A} and different protein regions.
Supports Figure 1.
Interaction between full-length CrMCA-II and serpin, an in vivo inhibitor of plant MCAs, was used as a positive control. The top row images are from Figure 1E and are included for comparison. AD, GAL4 activation domain; BD, GAL4 DNA-binding domain. p20, linker, and p10 encompass amino acids 1–153, 154–227, and 228–409 respectively. The results are representative example of two independent experiments.

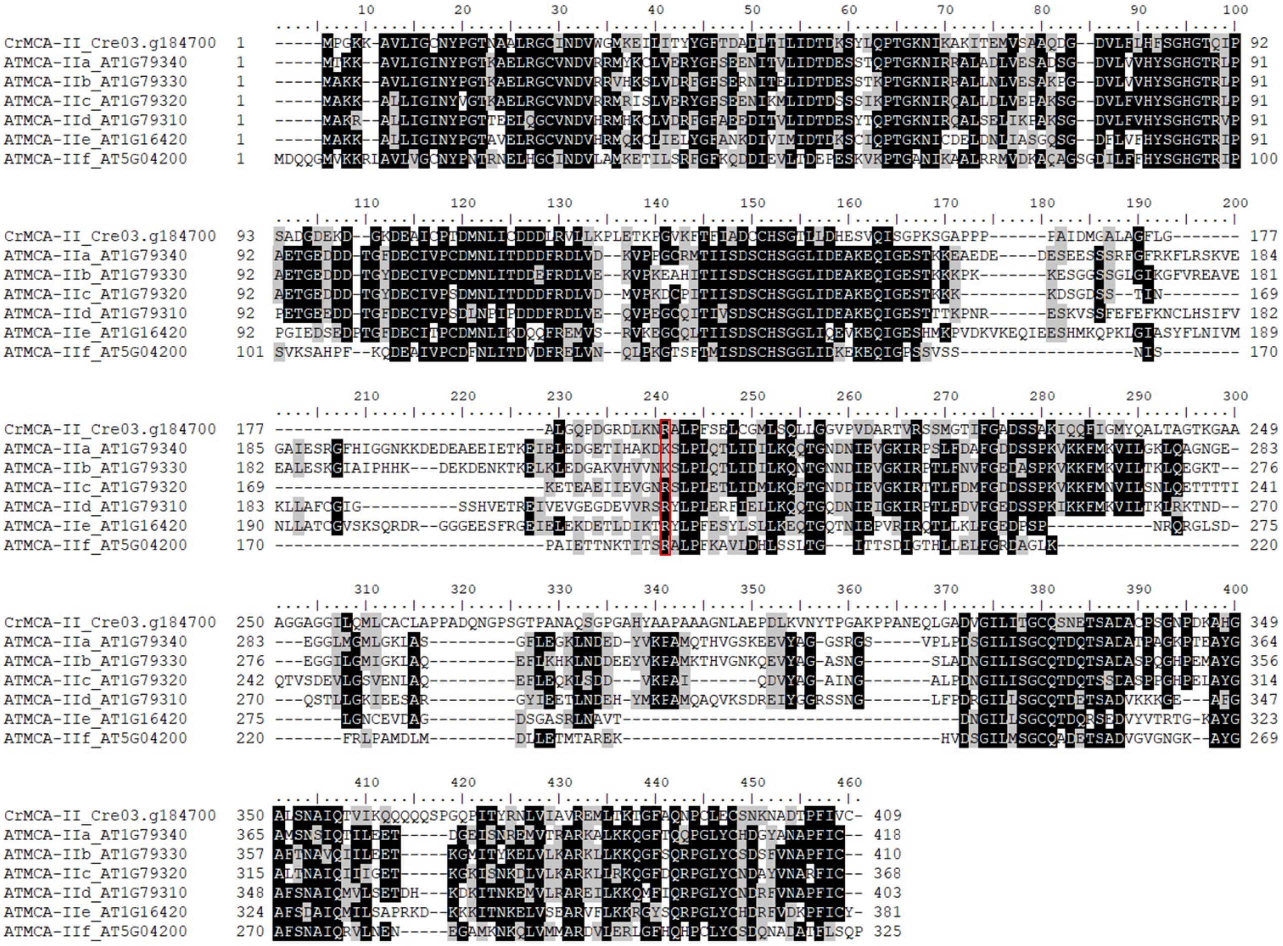

Supplemental Figure S6. Sequence alignment of type-II MCAs from Chlamydomonas and Arabidopsis.

Supports Figure 1.

The sequences were aligned using ClustalW (Larkin et al., 2007) and visualized by BioEdit (Hall, 1999). Identical amino acid residues are shaded in black and amino acid residues with similar traits are shaded in gray. Numbers on the left and right sides indicate the position of the first and last amino acids in the full-length protein sequence, respectively. The conserved Arg/Lys auto-cleavage site is denoted with a red rectangle.

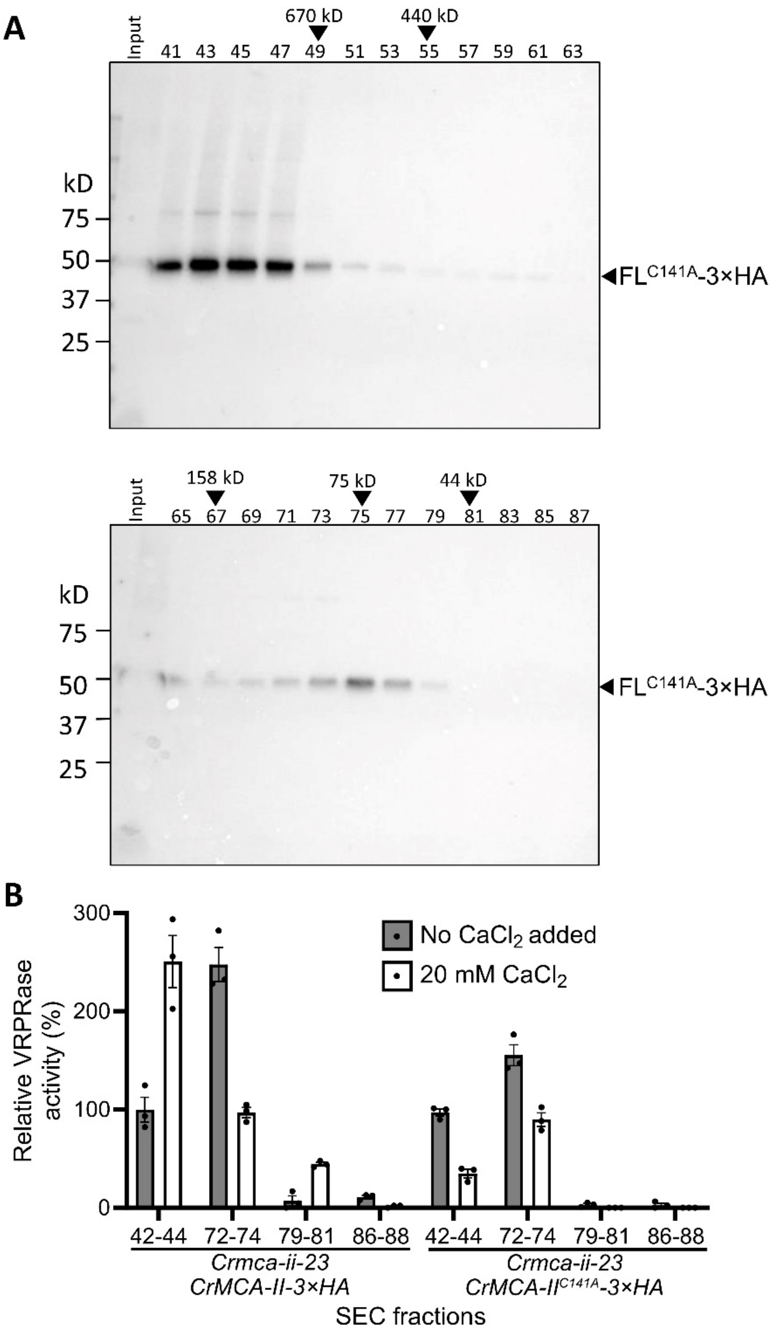

Supplemental Figure S7. Immunoblot and proteolytic activity analyses of SEC fractions from Chlamydomonas protein extracts.

Supports Figure 1.

(A) Immunoblot analysis of total protein extracts isolated from strain *Crmca-ii-23 CrMCA-II^{C141A}-3×HA* (see Figure 3) and fractionated by SEC. The procedure was as described for Figure 1H. Note the absence of CrMCA-II^{C141A}3×HA autoprocessing.

(B) Peptide (Ac-VRPR-AMC) cleavage assay of a subset of SEC fractions from strains *Crmca-ii23 CrMCA-II-3×HA* and *Crmca-ii-23 CrMCA-II^{C141A}-3×HA* under optimal buffer conditions, with or without addition of 20 mM $CaCl_2$. The fractions 42–44, 72–74, 79-81 and 86–88 correspond to megadalton assemblies, dimer, monomer and a fragment smaller than p10, respectively. Note the presence of a basal level of cell-derived Ca^{2+} in the assay. Note also background VRPRase activities in SEC fractions from strain *Crmca-ii-23 CrMCA-II^{C141A}-3×HA* devoid of active CrMCA-II. These background activities were subtracted from the activities in the respective fractions from strain *Crmca-ii-23 CrMCA-II-3×HA* to generate Figure 1I. The activity of fractions 42–44 was set to 100%. Data represent the means ± SEM of triplicate measurements.

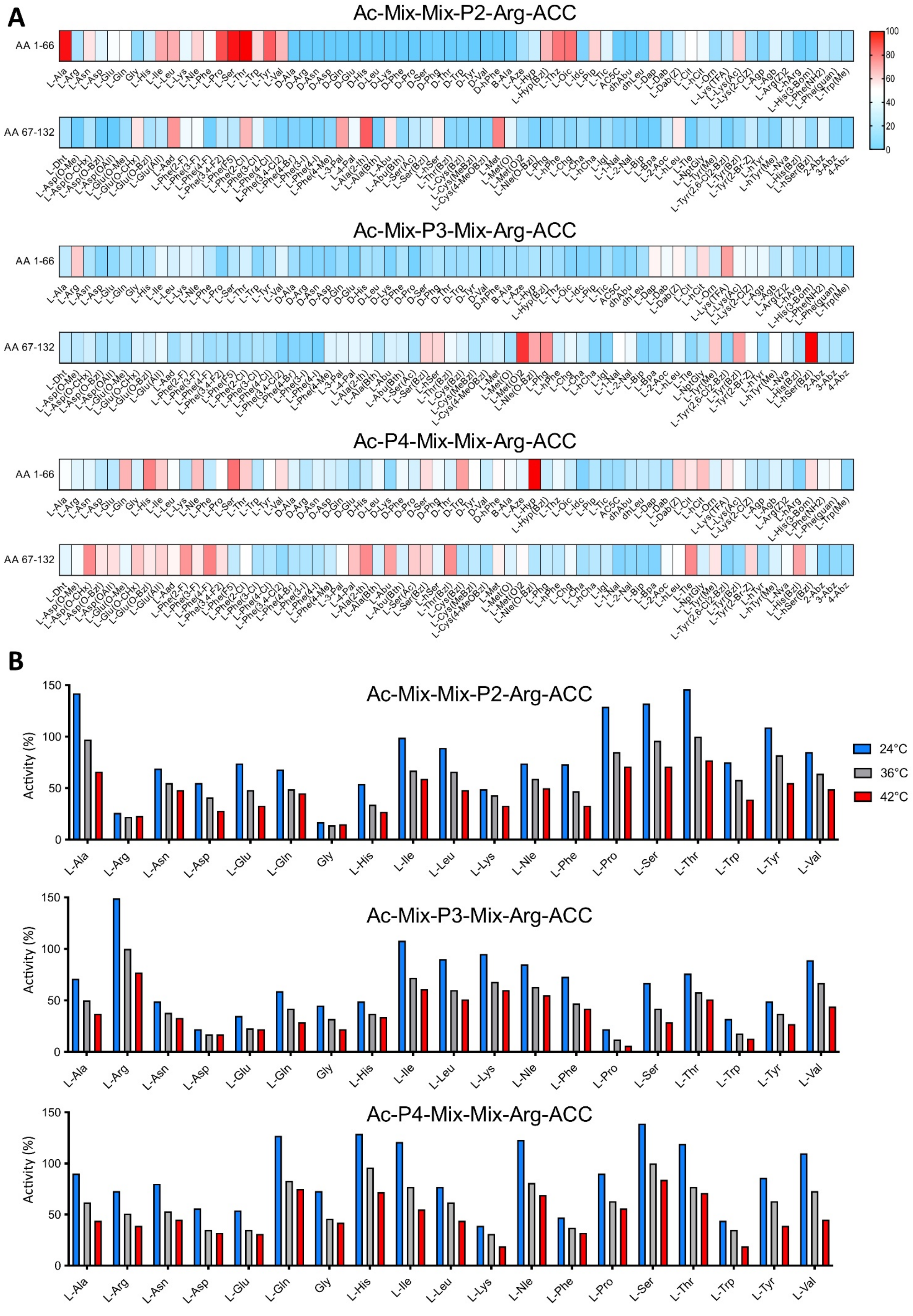
A
Ac-Mix-Mix-P2-Arg-ACC
AA 1-66
AA 67-132
Ac-Mix-P3-Mix-Arg-ACC
AA 1-66
AA 67-132
Ac-P4-Mix-Mix-Arg-ACC
AA 1-66
AA 67-132
100
80
60
40
20
0
B
Ac-Mix-Mix-P2-Arg-ACC
Ac-Mix-P3-Mix-Arg-ACC
Ac-P4-Mix-Mix-Arg-ACC
Activity (%)
150
100
50
0
24°C
36°C
42°C
L-Ala
L-Arg
L-Asn
L-Asp
L-Glu
L-Gln
Gly
L-His
L-Ile
L-Leu
L-Lys
L-Nle
L-Phe
L-Pro
L-Ser
L-Thr
L-Trp
L-Tyr
L-Val

Supplemental Figure S8. Substrate specificity of rCrMCA-II in the P4–P2 positions.
Supports Figure 2.
(A) Catalytic preferences of rCrMCA-II as determined using the P1-Arg HyCoSuL library, presented as heatmaps. The x-axis shows abbreviated amino acids (from 1 to 132), and the heatmaps display the relative activity of each substrate adjusted to the best recognized amino acid in each position marked in red.
(B) Effect of temperature on the catalytic preferences of rCrMCA-II. The analysis was performed at three different temperatures using the P1-Arg HyCoSuL library composed of only natural amino acids. The highest activity of each sub-library (P2, P3, P4) screening at 36°C was set to 100%.
Each sub-library (P2, P3, P4) screening was performed in triplicate under optimal buffer conditions and the rate of substrate hydrolysis is represented as average. SD for each measurement was below 15%.

Ac-Val-Asp-Pro-Arg-ACC

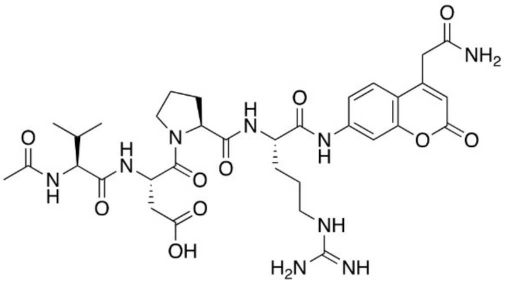

Ac-His-Agp-Met-Arg-ACC

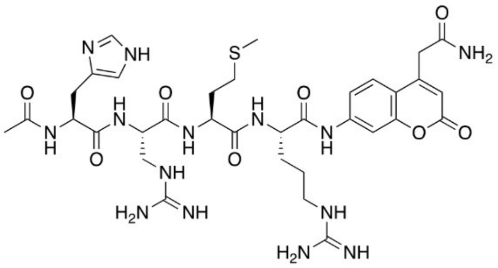

Ac-Val-Arg-Pro-Arg-ACC

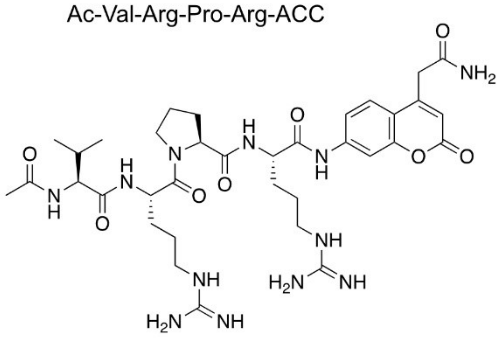

Ac-His(Bzl)-Agp-Ala-Arg-ACC

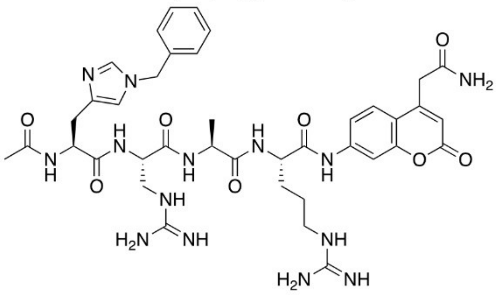

Ac-Ser-Arg-Thr-Arg-ACC

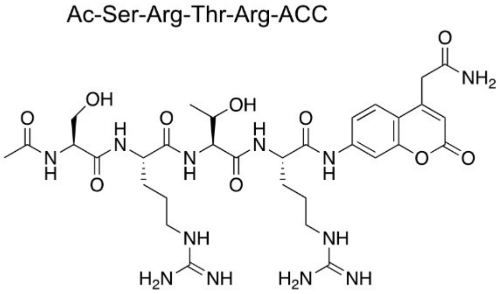

Ac-His-Dap-Ala-Arg-ACC

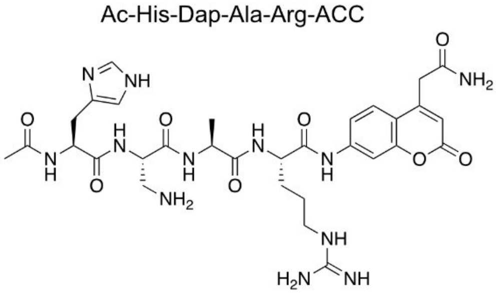

Ac-His-Arg-Thr-Arg-ACC

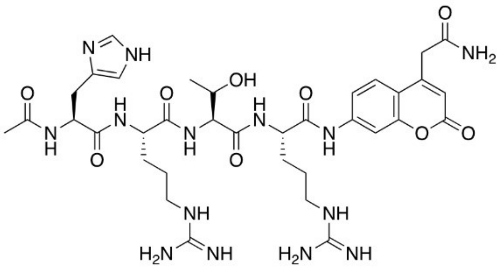

Ac-His(Bzl)-Arg-Thr-Arg-ACC

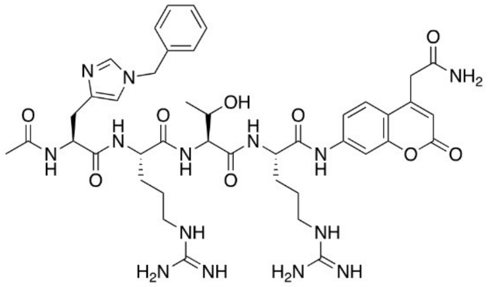

Ac-hP-hS(Bzl)-Thr-Arg-ACC

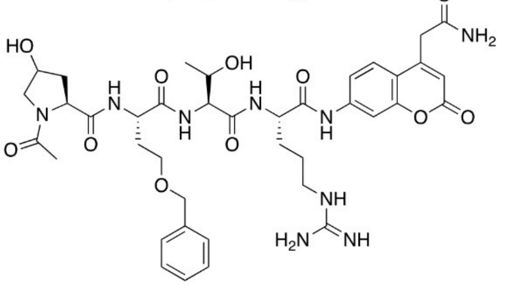

Ac-Ser-Agp-Thr-Arg-ACC

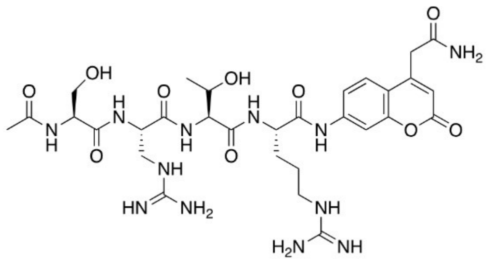

Ac-Ser-Met(O_2)-Thr-Arg-ACC

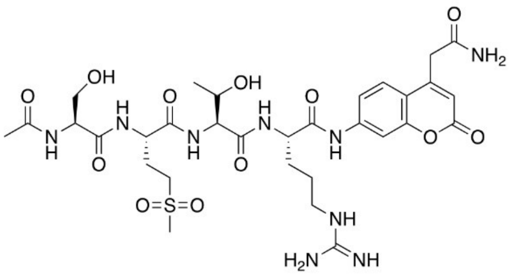

Ac-His(Bzl)-hSer(Bzl)-Thr-Arg-ACC

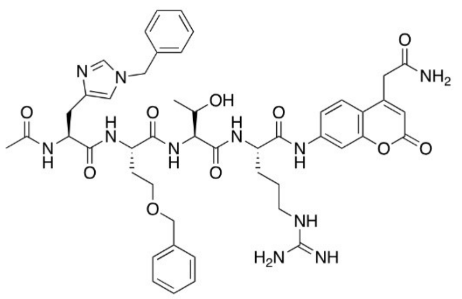

Ac-Ser-Tyr(Bzl)-Thr-Arg-ACC

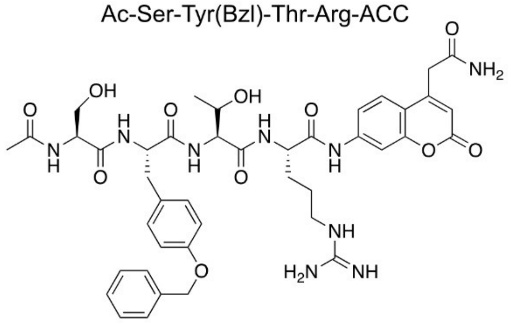

Ac-His-hSer(Bzl)-Met-Arg-ACC

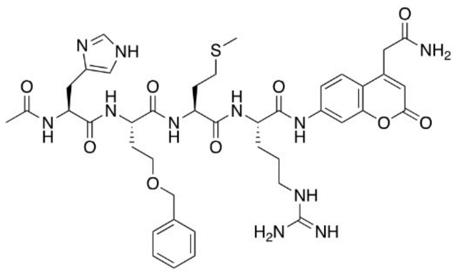

Ac-Ser-hS(Bzl)-Pro-Arg-ACC

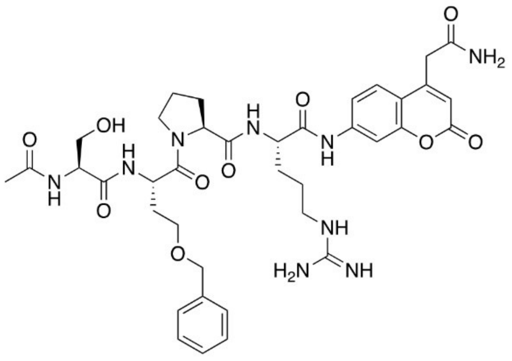

Ac-Ser-hSer(Bzl)-Thr-Arg-ACC

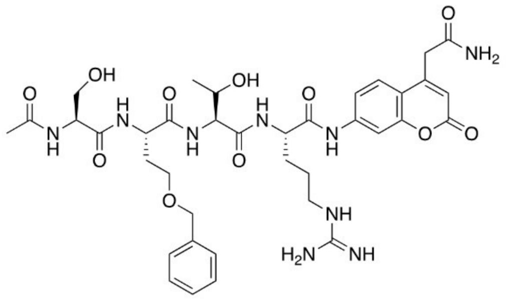

Ac-His(Bzl)-Dap-Met-Arg-ACC

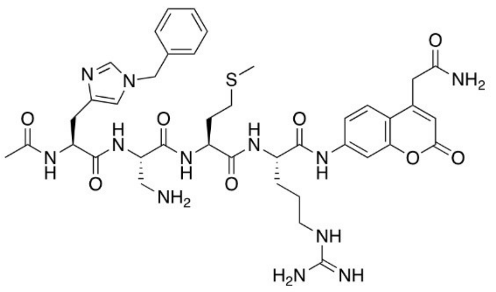

Supplemental Figure S9. Structures of ACC-labeled tetrapeptide substrates for CrMCA-II.
Supports Figure 2.

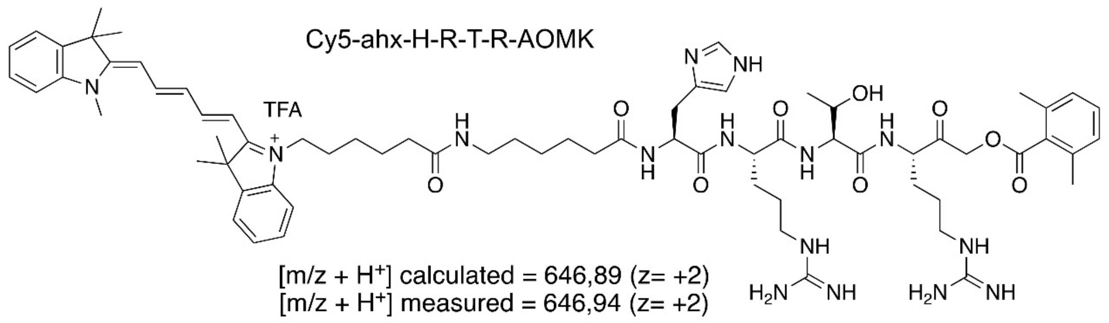

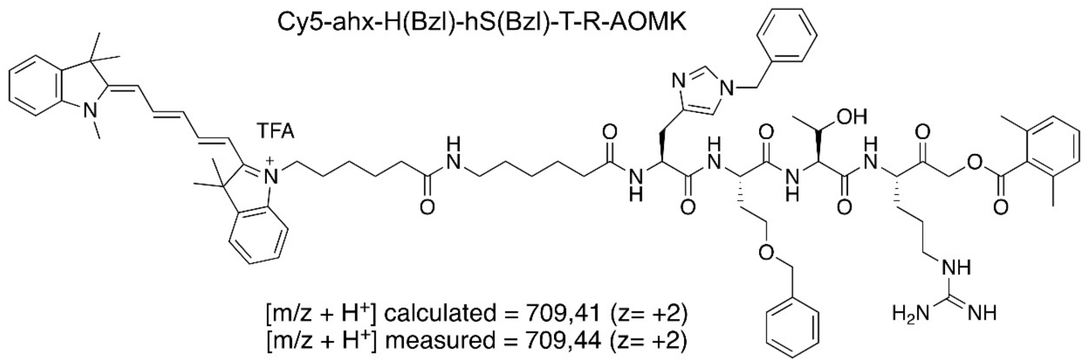

Supplemental Figure S10. Structures and MS data of Cy5-labeled ABPs for CrMCA-II.
Supports Figure 2.

UVM4	GCCAAGATCACGGAGATGGTGAGCGCCGCCCAGGACGGGGATGTGCTGTTCCTGCACTTCAGCGGCCACGGAACCCAGATCCCCAGCGCAGACGGCGACGAGAAGG
Crmca-ii-9	GCCAAGATCACGGAGATGGTGAGCGCCGCCCAGGACGGGGATGTGCTGTTCC-paro cassette--CGGAACCCAGATCCCCAGCGCAGACGGCGACGAGAAGG
Crmca-ii-23	GCCAAGATCACGGAGATGGTGAGCGCCGCCCAGGAC---------paro cassette----------CGGAACCCAGATCCCCAGCGCAGACGGCGACGAGAAGG

GTGCTGTTCCTGCACTTCAG: gRNA targeting site
CGG: protospacer adjacent motif (PAM)
paro cassette: PSAD promoter + *AphVIII* gene + PSAD terminator(~1370 bp)

Supplemental Figure S11. Sequence alignment of a part of exon 4 of *CrMCA-II* genomic DNA from control (UVM4) and mutant (*Crmca-ii-9* and *Crmca-ii-23*) strains.
Supports Figure 3.
Paro cassette, paromomycin resistance cassette.

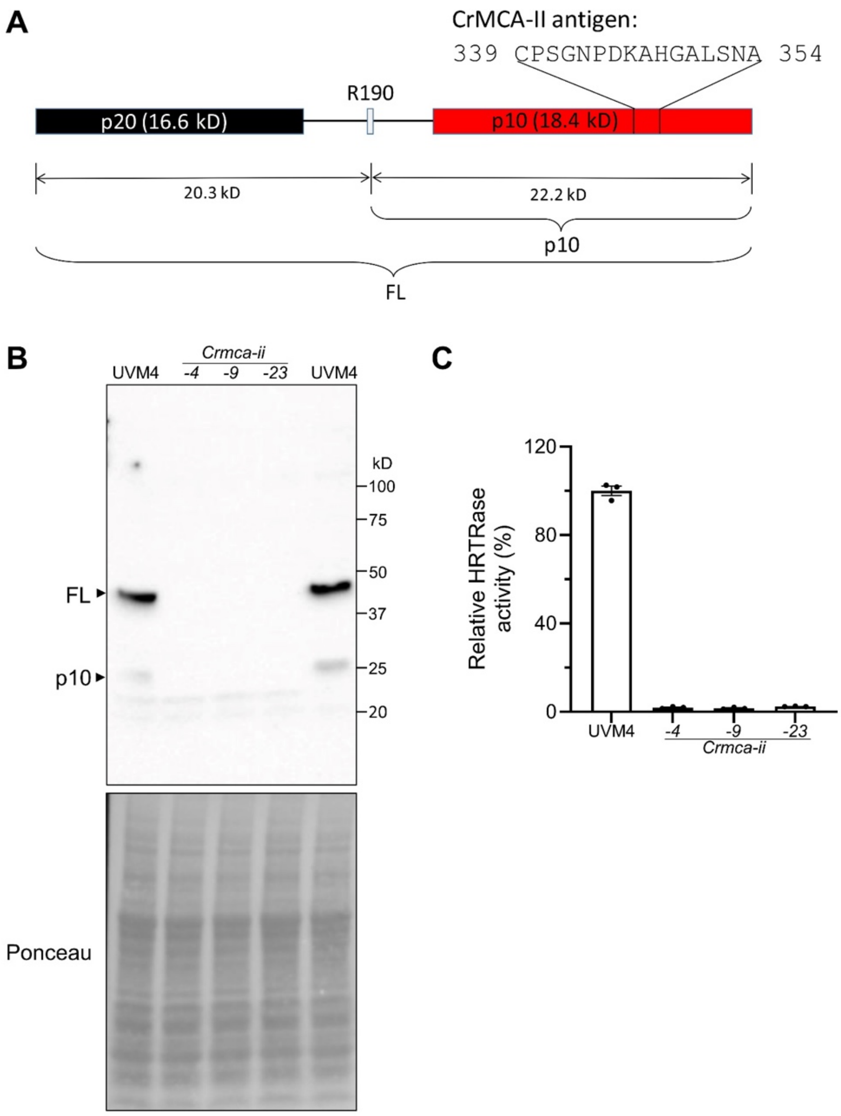

Supplemental Figure S12. Characterization of *Crmca-II* mutants using a specific anti-CrMCA-II antibody and substrate cleavage assay.

Supports Figure 3.

(A) Diagram of CrMCA-II domain organization showing location and sequence of the peptide used as antigen for generating the anti-CrMCA-II antibody. While p10 region of CrMCA-II is 18.4 kD, the lower band detected on immunoblots of total protein extracts with the anti-CrMCA-II antibody corresponds to a 22.2-kD fragment that includes the p10 and a part of the linker region generated via the auto-cleavage at Arg-190.

(B) Representative immunoblot of total protein extracts from UVM4 and *Crmca-ii* mutants with the anti-CrMCA-II antibody recognizing zymogen (full-length, FL) and the 22.2-kD fragment (indicated as p10). Ponceau staining of the PVDF membrane was used as a loading control.

(C) Ac-HRTR-ACC cleavage assay using cell lysates from UVM4 and *Crmca-ii* mutants. Data represent the means ± SEM of triplicate measurements.

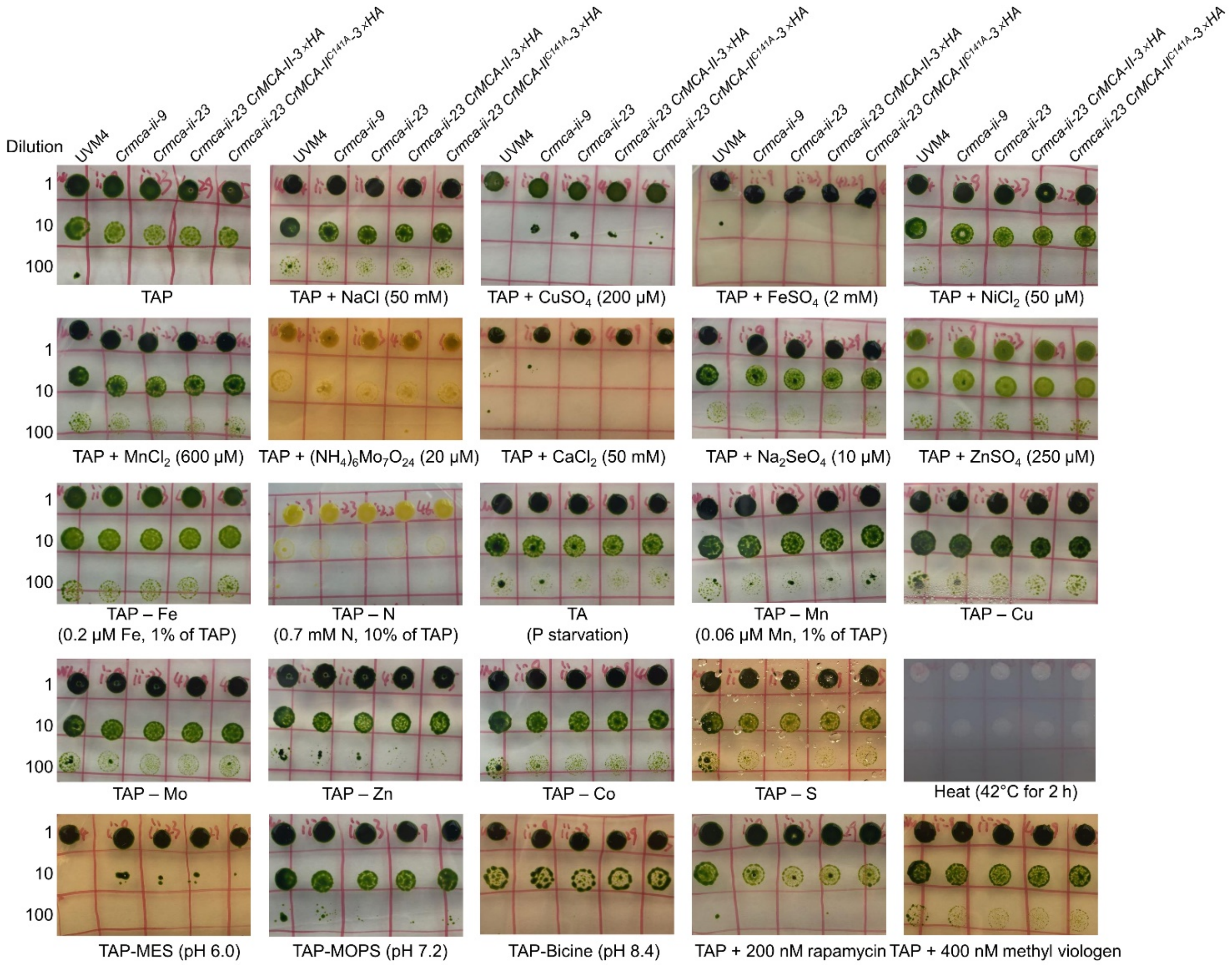

Supplemental Figure S13. Phenotyping of UVM4, *Crmca-ii* mutant (*Crmca-ii-9* and *Crmca-ii-23*) and complementation (*Crmca-ii-23 CrMCA-II-3×HA* and *Crmca-ii-23 CrMCA-II^{C141A}-3×HA*) strains grown on plates under various nutrient and environmental conditions.

Supports Figure 4.

The numbers of log-phased cells plated from each strain were 1×10^5, 1×10^4, and 1×10^3 for dilutions 1, 1:10 and 1:100, respectively. The plates were imaged after 20 d of growth.

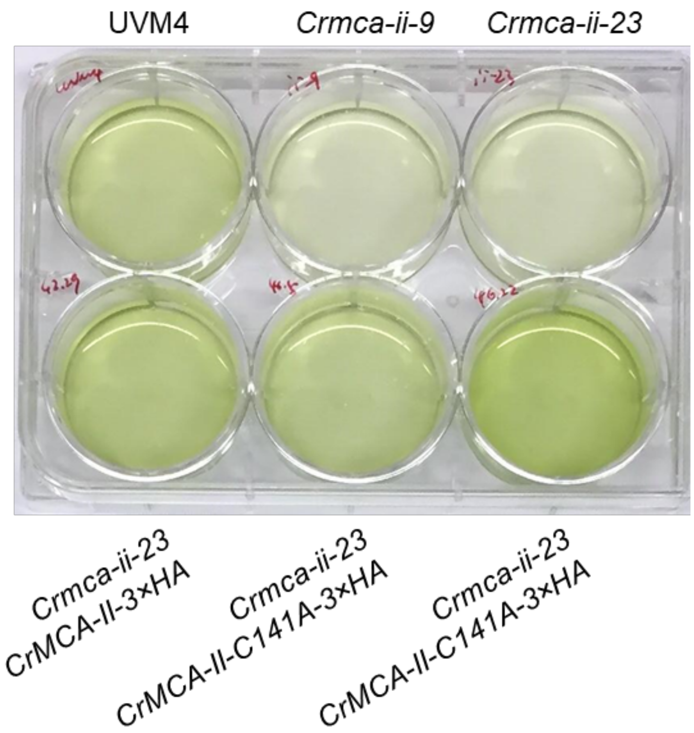

Supplemental Figure S14. Algal strains 6 d after recovery from a 2-h HS treatment at 42°C.
Supports Figure 4.
One hundred µL cells at log phase (5-6×10^6 cells mL $^{-1}$) were heat-treated for 2 h at 42°C. Fifty µL culture were then inoculated into 3 mL fresh TAP medium for recovery and OD750 measurements (Figure 4A).

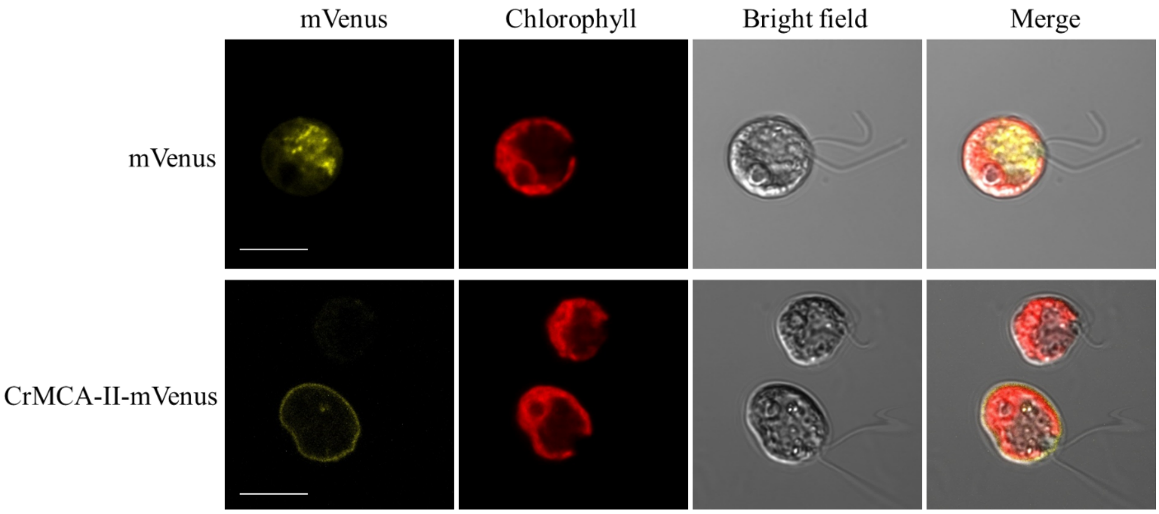

Supplemental Figure S15. Subcellular localization of mVenus and CrMCA-II-mVenus in strain CC-4533 under normal conditions.
Supports Figure 5.
Scale bars, 10 µm.

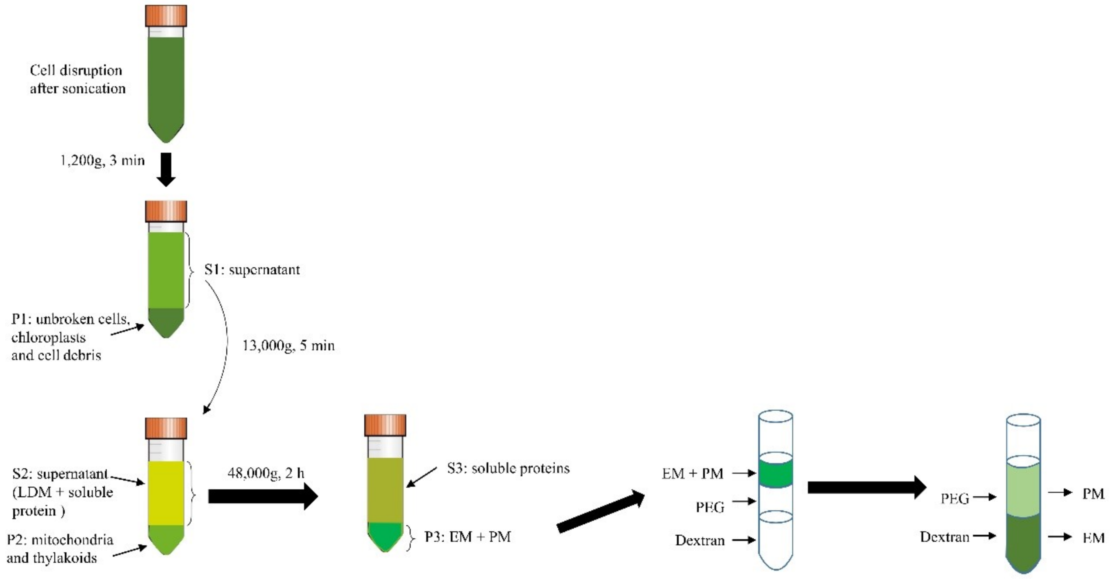

Supplemental Figure S16. Procedure for subcellular fractionation used for CrMCA-II and H$^+$ATPase immunoblot analysis shown in Figure 5B and 5D.

Supports Figure 5.

EM, endomembrane fraction; PM, plasma membrane fraction; LDM, low-density membranes; PEG, polyethylene glycol.

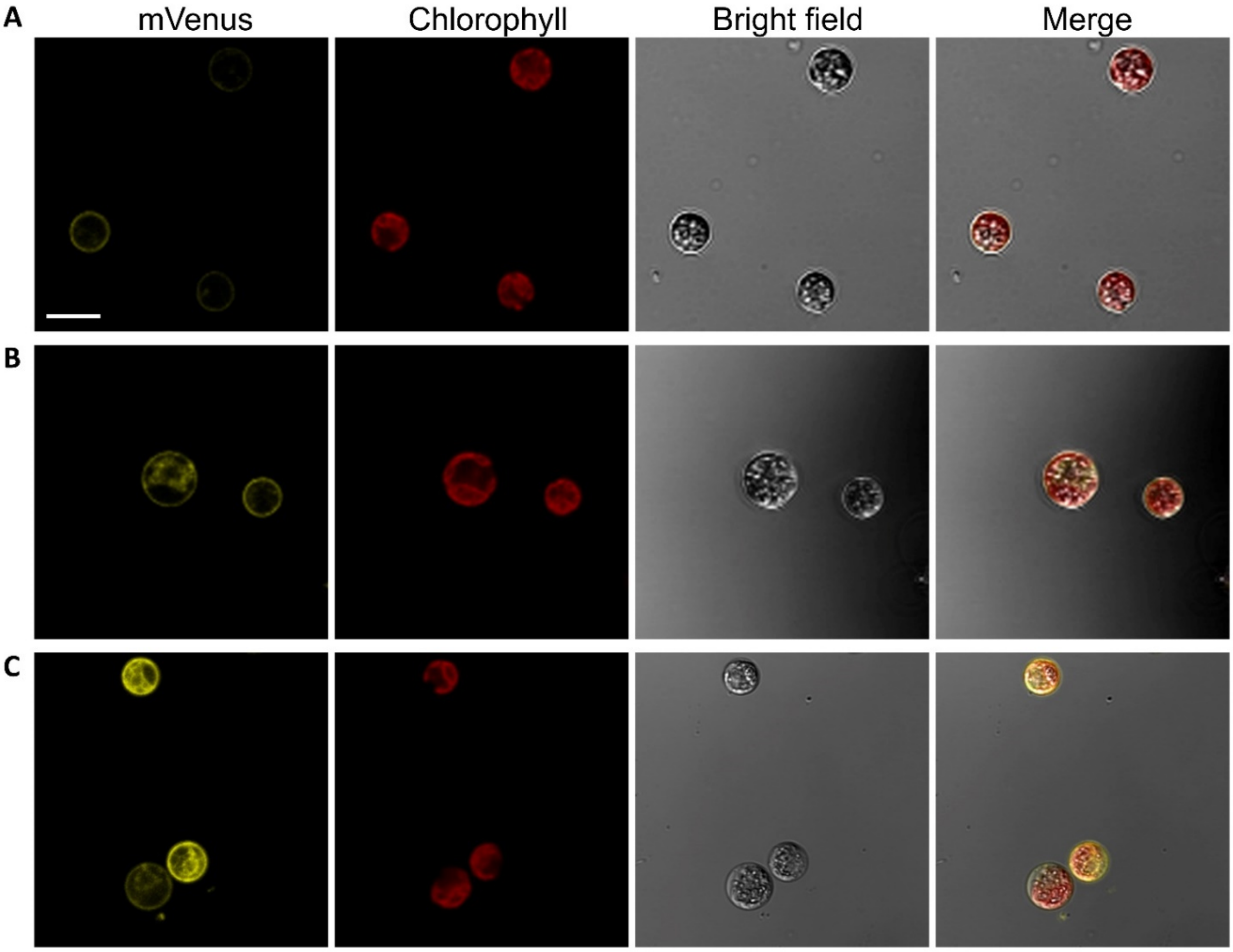

Supplemental Figure S17. Confocal microscopy analysis of CrMCA-II localization.
Supports Figure 5.
(**A, B**) Localization of CrMCA-II^{C141A}-mVenus (yellow) in strain UVM4 before (**A**) or after (**B**) HS at 42°C for 60 min.
(**C**) Localization of CrMCA-II-mVenus under HS in the cells treated with 35 μM cycloheximide.
Scale bar, 10 μm.

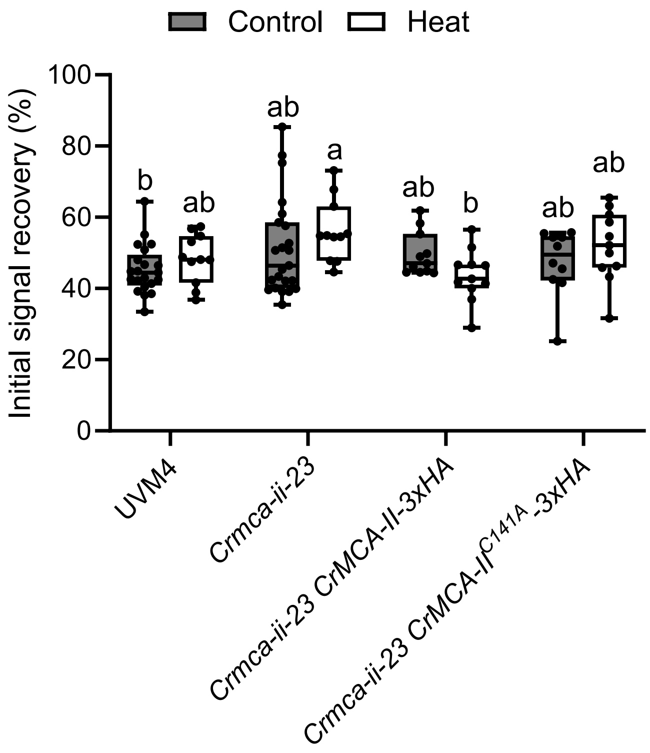

Supplemental Figure S18. FRAP analysis.
Supports Figure 5.
Initial signal recovery of DiOC6(3) after photo-bleaching of cells from the indicated strains grown under control conditions (23°C, Control) or HS (39°C for 60 min, Heat). Different letters indicate significant differences at $P < 0.05$, as determined by a two-way ANOVA with Tukey's honest significant difference test. The upper and lower box boundaries respectively represent the first and third quantiles, horizontal lines denote the median, and whiskers indicate the highest and lowest values. The data are from three independent biological experiments, each including at least 3 measurements (individual cells).

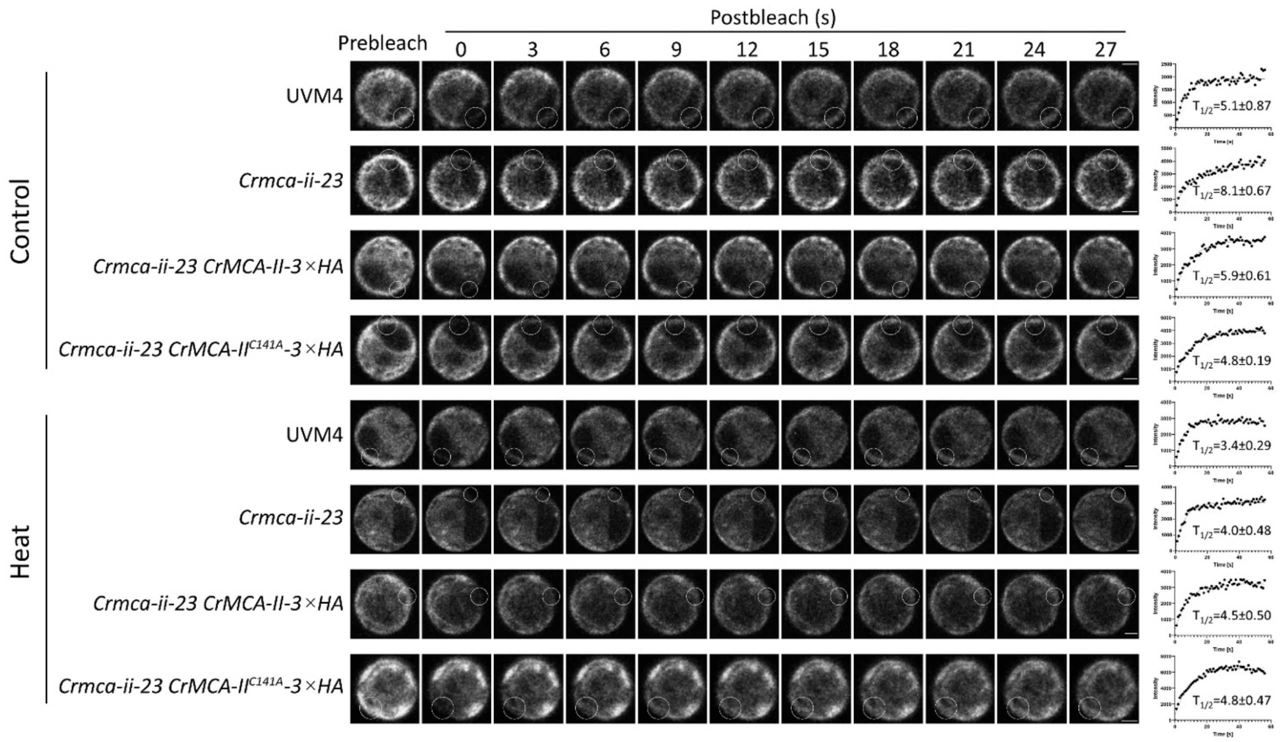

Supplemental Figure S19. Selected time frames from FRAP analysis shown in Figure 5G.
Supports Figure 5.
The circles indicate the bleached foci. Shown on the right are FRAP curves and signal recovery values ($T_{1/2}$; means ± SEM of three independent biological experiments, each including at least 3 measurements). The signal intensity at the postbleach time point 0 was set to zero. Scale bars, 2 µm.

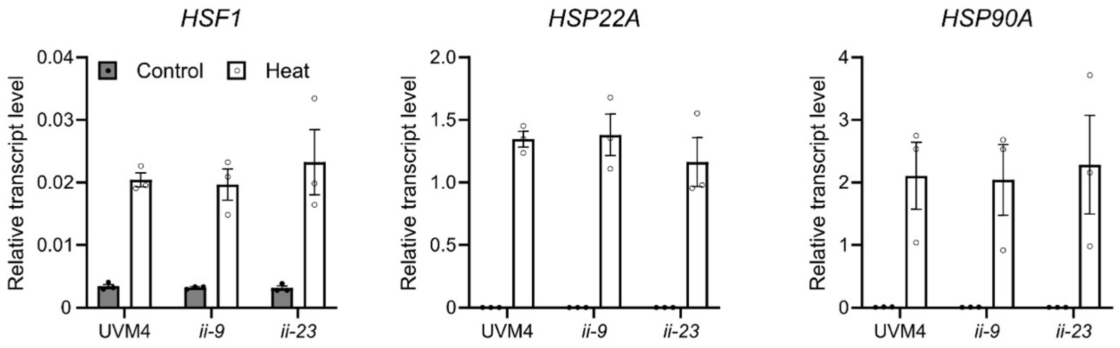

Supplemental Figure S20. RT-qPCR analysis of HS responsive gene expression.
Supports Figures 4 and 5.
Relative transcript levels of *HSF1*, *HSP22A* and *HSP90A* in UVM4 and *Crmca-ii* mutants (*Crmca-ii-9*, *Crmca-ii23*) before (Control) and after 42°C HS for 30 min (Heat). *RACK1* was used as internal control and its transcript level was set to 1. Data represent the means ± SEM of three independent biological experiments.

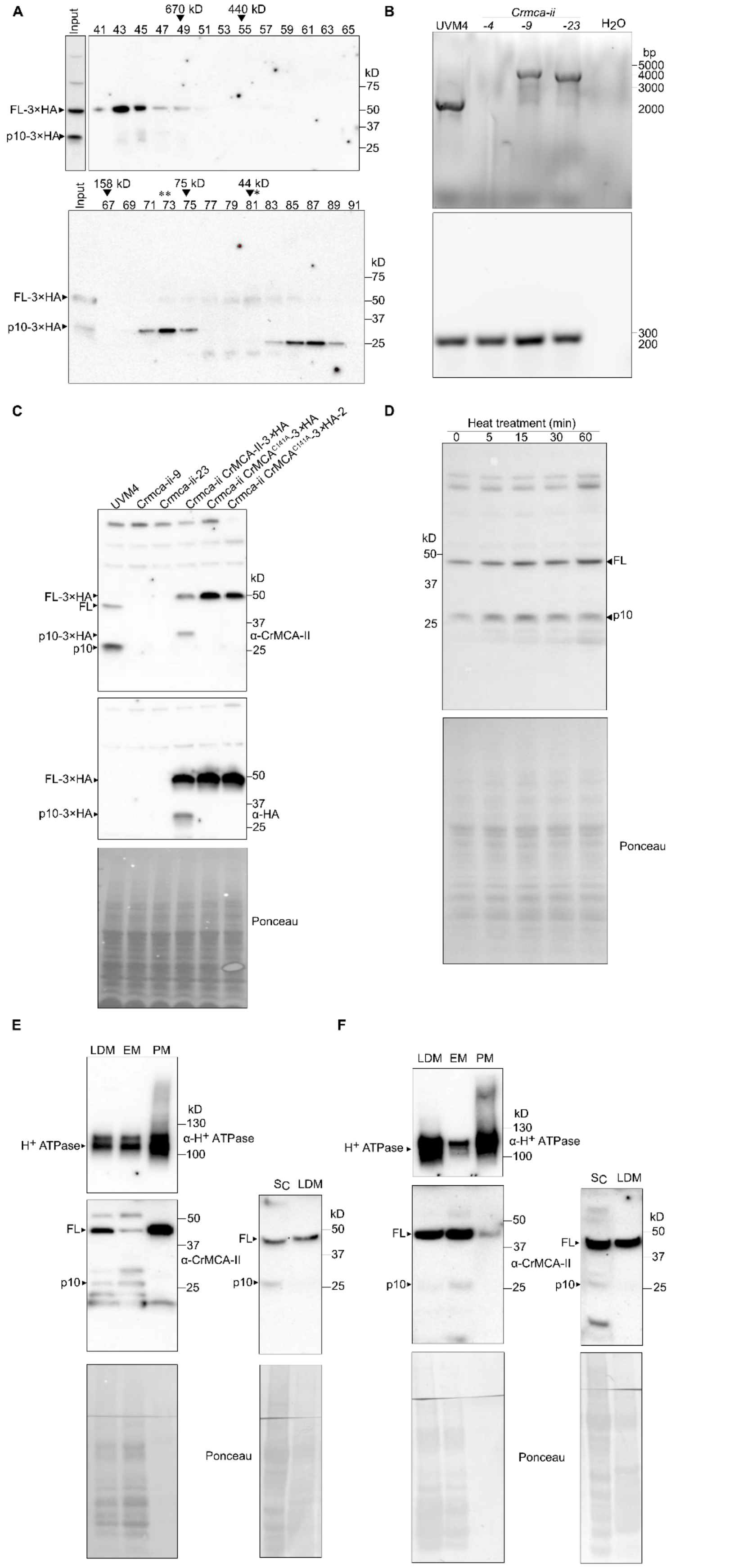

Supplemental Figure S21. Uncropped gel and membrane images.
(A) Figure 1H; **(B)** Figure 3B; **(C)** Figure 3E; **(D)** Figure 4C; **(E)** Figure 5B; **(F)** Figure 5D

Supplemental Table S1. Activity-based probes generated and used in this study.

Name	Reference
Cy5-ahx-HRTR-AOMK	This study
Cy5-ahx-H(Bzl)-hS(Bzl)-TR-AOMK	This study
